# WS_2_ Optoelectronic Memristive Reservoir Enabling Ultra‐Low‐Power, Multi‐Task, and Environmentally Stable Neuromorphic Computing

**DOI:** 10.1002/advs.75318

**Published:** 2026-04-17

**Authors:** Dayanand Kumar, Hanrui Li, Divyanshu Divyanshu, Dhananjay D. Kumbhar, Manoj Kumar Rajbhar, Amit Singh, Abdul Momin Syed, Selma Amara, Gianluca Setti, Nazek El‐Atab

**Affiliations:** ^1^ Smart, Advanced Memory Devices and Applications (SAMA) Laboratory Electrical and Computer Engineering Computer Electrical Mathematical Science and Engineering Division King Abdullah University of Science and Technology (KAUST) Thuwal Kingdom of Saudi Arabia; ^2^ Integrated Intelligent Systems (I2S) Lab Electrical and Computer Engineering Computer Electrical Mathematical Science and Engineering Division King Abdullah University of Science and Technology (KAUST) Thuwal Kingdom of Saudi Arabia

## Abstract

Energy‐efficient visual and speech processing is essential for edge intelligence, yet conventional silicon‐based chips suffer from high power consumption. Here, we report a WS_2_/Zinc–Tin–Oxide (ZTO)‐based optoelectronic reservoir computing (RC) system that uniquely integrates sensing, memory, and computation within a single compact device to emulate diverse biological functions. The WS_2_/ZTO memristive RC achieves strong performance, with ∼94% accuracy on N‐MNIST, ∼93% in motion perception, and ∼89% in speech recognition within only 30 training epochs, while consuming ultra‐low energy of ∼25.5 fJ/spike. Raw inputs are converted into spike trains to preserve temporal dynamics: motion data from inter‐frame differences, FSDD waveforms reshaped into spike‐like signals, and N‐MNIST reconstructed directly from the address‐event representation format. The system maintains reliable operation under 95% relative humidity, highlighting excellent environmental stability. Distinctively, the WS_2_/ZTO memristor serves simultaneously as sensor and hardware reservoir, exploiting volatile and nonlinear dynamics for direct temporal input decoding. Validation on N‐MNIST further shows 95% accuracy with minimal training energy. In addition, the device demonstrates endurance over 1.5 million cycles and supports synaptic features including excitatory postsynaptic current, short‐term and long‐term plasticity, and photonic paired‐pulse facilitation. This work establishes a humidity‐resilient, ultra‐low‐power WS_2_/ZTO in‐sensor RC platform, advancing neuromorphic processing for next‐generation edge technologies.

## Graphical abstract

WS_2_‐based in‐memory sensing reservoir computing integrates sensing, memory, and computation in one compact device. It achieves ∼94% N‐MNIST, ∼93% eye motion perception, and ∼89% speech recognition with ultra‐low energy (∼25.5 fJ/spike). The system shows stability at 95% humidity, endurance over 1.5M cycles, and supports synaptic plasticity, enabling energy‐efficient, humidity‐resilient neuromorphic processing for next‐generation edge intelligence.

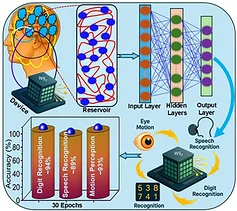

## 1 Introduction

The human retina serves not only as a light sensor but as a rapid interpreter of dynamic signals, enabling precise learning in the subsequent regions of the visual cortex [1, 2, 3, 4]. This synergistic interplay between the retina and visual cortex establishes the foundation for the brain's capacity to manage multiple tasks concurrently [5, 6, 7], a crucial aspect for advancing machine vision [8]. On the other hand, the machine vision technology enables the inspection and analysis of the surrounding environment using image sensors integrated with processing units [9]. Recent advancements in image sensing and artificial intelligence (AI) technologies have significantly enhanced the efficient generation of digital images from the physical world and the interpretation of these acquired images [10].

In terms of hardware, the development of non‐von Neumann‐based semiconductor architectures that mimic the biological functions of neurons and synapses within semiconductor devices, in line with the principles of artificial synaptic electronics, has been crucial for enhancing power efficiency and computational speed. This highlights the need for advancing multimodal AI technologies that seamlessly integrate computing functions across sensory systems. Such convergence is increasingly vital due to the growing demand for real‐time communication platforms and intelligent mobility solutions. In contrast, conventional silicon‐based vision chips, which separate sensing, processing, and memory functions, encounter significant challenges, including delays and increased energy consumption due to the frequent data transfers between these components. The stepwise analog‐to‐digital conversions further constrain their energy efficiency [11, 12, 13]. Moreover, standard deep‐learning frameworks, such as recurrent neural networks that handle time‐based signals [14], often demand extensive training for particular applications, a demand that is unfeasible for devices on the edge with limited battery life and size constraints. In view of the algorithm, reservoir computing provides benefits of energy efficiency among fixed memory with sharing synaptic connections, which can be naturally used in power‐limited edge computing or dynamic temporal signal processing scenarios [15, 16]. Liquid state machine (LSM) encoder takes advantage of both Spiking neural network (SNN) and reservoir computing (RC), which is effective for capturing features in higher dimensional space and resembles the processes of visual cognition. However, it is still in progress to co‐design a device‐algorithm compatible bio‐inspired vision system, which combines the energy‐efficient sensing ability of artificial retina and affordable edge learning algorithms.

Extensive research has been dedicated to replicating the human retina and its sophisticated learning mechanisms, aiming to bridge the gap between biological and artificial vision systems. From a materials science perspective, 2D materials, composed of atomically thin layers, exhibit an array of exceptional properties, such as high carrier mobility, tunable bandgaps, and mechanical flexibility. These attributes position 2D materials at the forefront of the next generation of electronic devices, offering unprecedented opportunities to enhance the performance and functionality of neuromorphic systems and bio‐inspired technologies. The ability of these materials to integrate seamlessly into complex architectures underscores their potential to revolutionize future advancements in optoelectronics and cognitive computing [17, 18, 19]. In particular, the focus on layered transition metal dichalcogenides (TMDCs) has opened a wealth of opportunities for creating energy‐efficient electronics, advanced memory devices, and innovative computing architectures that benefit from reduced size [20, 21]. Recently, there's been a surge in research into nonvolatile devices crafted from 2D materials and their potential applications in the field of AI [22, 23, 24, 25]. Inorganic 2D semiconductors responsive to light, such as MoS_2_ with its defects and impurities [26], SnS featuring dual‐type defects linked to both Sn and S atoms [15], layered black phosphorus with oxidation‐induced defects [27], perovskite quantum dots known for their significant photogating effects [28, 29], the h‐BN/WSe_2_ heterostructure that excels in electron trapping and de‐trapping [30], are all notable for their unique properties.

Among these, WS_2_ stands out as one of the most promising materials for artificial retinas, attributed to its lower relative mass alongside its exceptional thermal and chemical durability, marking it as a superior choice compared to other TMDCs [31, 32]. Additionally, WS_2_’s effectiveness is enhanced by its abundance of crystal defects, including grain boundaries, vacancies, and impurities, which are crucial for enabling the resistive switching (RS) effect [31, 33, 34, 35]. In the past few years, minimal research has been conducted on WS_2_‐based synaptic devices for neuromorphic computing systems [21, 31, 36, 37, 38, 39, 40, 41]. To our knowledge, this is the first demonstration of ultra‐low power and humidity‐resilient in‐memory sensor RC using WS_2_ optoelectronic memristors for multitask learning and event‐based data encoding.

In this study, a WS_2_‐based dynamic in‐memory sensor RC system is introduced, integrating sensing, memory, and computation within a single compact device to replicate multiple biological functions. The WS_2_ memristive RC demonstrates strong performance across vision, tracking, and speech recognition tasks with exceptionally low optical energy consumption of ∼25.5 fJ/spike. The device also exhibits remarkable environmental robustness, maintaining reliable operation under high humidity conditions owing to a zinc–tin–oxide encapsulation layer. Unlike conventional approaches, the WS_2_ memristor functions simultaneously as both sensor and hardware reservoir, leveraging volatile and nonlinear dynamics to directly process temporal inputs. Its reservoir encoding capability is further validated on event‐driven datasets with minimal training energy requirements. In addition, the system shows reproducible electrical and optical characteristics, AC endurance exceeding 1.5M cycles, and advanced optoelectronic synaptic responses, including excitatory photo‐synaptic current (EPSC), short‐term plasticity (STP), long‐term plasticity (LTP), and photonic paired pulse facilitation (PPF). Collectively, these results establish the first humidity‐resilient, ultra‐low‐power, and multifunctional WS_2_ in‐sensor RC platform, opening new opportunities for energy‐efficient, event‐driven neuromorphic processing in next‐generation edge technologies. Table 1 presents a comparison of the optoelectronic characteristics of the WS_2_‐based optoelectronic synaptic device demonstrated in this work with those of previously reported single‐layer and bilayer WS_2_‐based synaptic devices. The results clearly indicate that our device exhibits superior optoelectronic synaptic performance, along with multitasking capabilities, compared to previously reported devices. We have also compared our device with other previously reported TMD‐based optoelectronic devices, as summarized in Table S1.

**TABLE 1:** Comparison of previously reported single‐layer and bilayer WS_2_ based synaptic devices with the WS_2_‐based device demonstrated in this work.

Material	Operation	Endurance (cycles)	Synaptic function	Energy consumption	Applications	Accuracy	Ref.
ZrO_2_/WS_2_	Electrical	∼10^9^	PPF/STDP	NA	Digit Recognition	87%	[42]
WS_2_/In_2_Se_3_	Optical	NA	P/D	7.7 × 10^−18^J	Digit Recognition	∼80%	[43]
Fe_2_O_3_/WS_2_	Electrical	∼10^5^	PPF/PPD	7.2 × 10^−^^14^ J	Image Recognition	∼94%	[44]
HfO_2_/WS_2_	Electrical	2 × 10^3^	PPF/PPD	NA	Digit Recognition	∼94%	[45]
PdSe_2_/WS_2_	Optical	NA	PPF	NA	Image Recognition/ Visual Perception	∼80%	[46]
WS_2_	Electrical	NA	STDP/PPF	2.998 × 10^−13^J	NA	NA	[37]
WS_2_	Optical	NA	PPF/STDP	1.5 × 10^−13^J	Image Recognition	∼93%	[47]
WS_2_	Electrical	400	LTP/LTD	∼10^−12^J	Digit recognition	∼94%	[21]
WS_2_	Electrical + Optical	1.5 × 10^6^	LTP/LTD/STP /LTP/STM/ LTM/PPF	2.5 × 10^−^^14^ J	Digit Recognition	∼94%	This Work
					Motion Perception	∼93%	
					Speech Recognition	∼89%	
					Reservoir Encoder	∼95%	

## 2 Results

**FIGURE 1:**
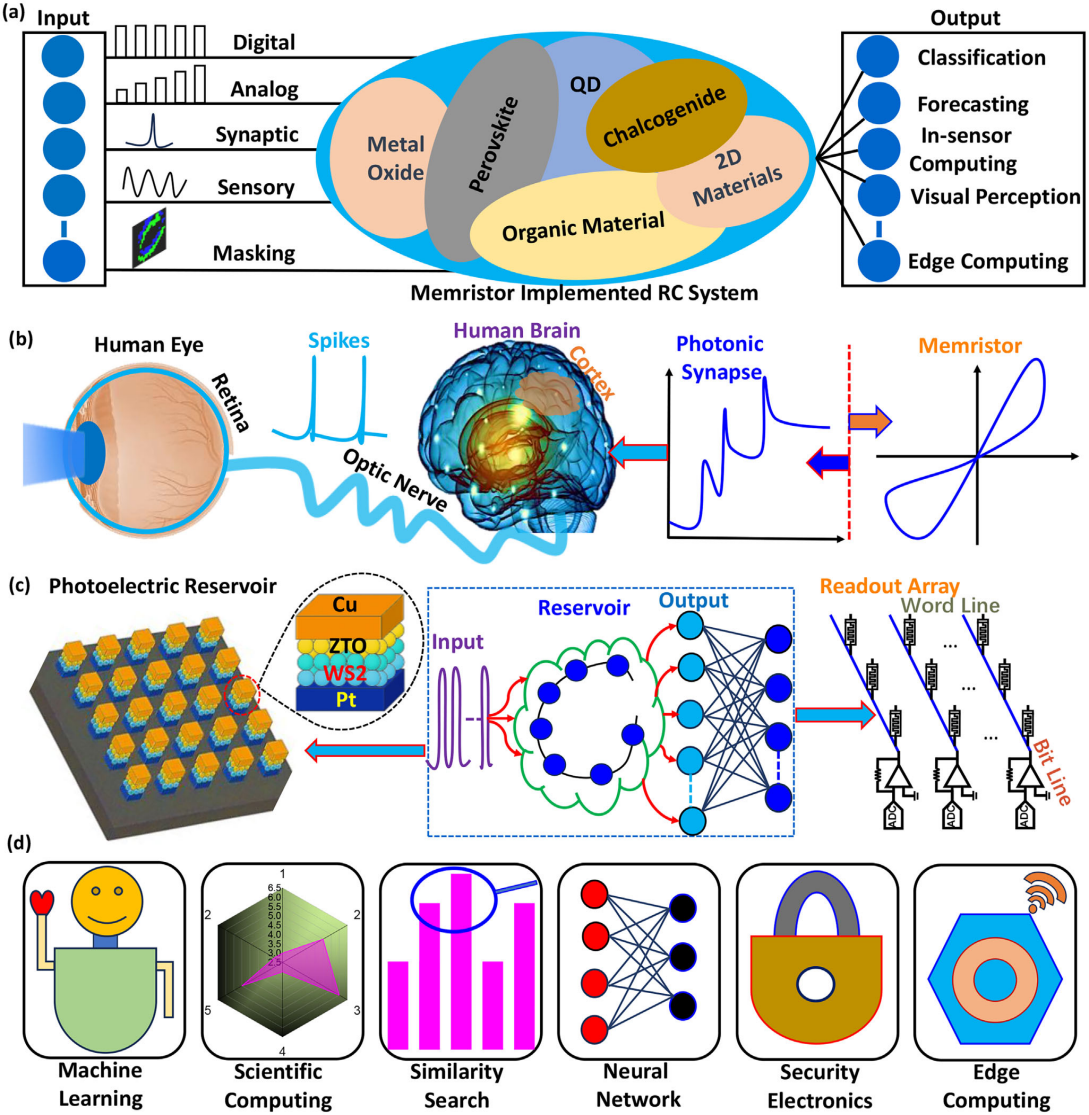
(a) The schematic illustration of reservoir computing (RC) using memristors highlights a system designed to process diverse input types, such as digital, analog, synaptic, sensory, and pattern‐masking inputs. (b) Schematic diagram of the human visual recognition system, consisting of the retina, optical neurons, and the visual cortex in the human brain. (c) The schematic of proposed RC system based on ZTO/WS_2_, utilizes optical synapses as the input layer of the reservoir, with a memristor device array forming the readout network. Detailed in the inset within a dashed box is the abstract photoelectronic RC system. In this system, original optical information is fed into the photoelectronic reservoir, where the inputs are nonlinearly transformed into feature outputs through the PPC effect. Subsequently, the memristor array receives these outputs from the reservoir and conducts readout training. (d) Schematic illustrating the advancements in this domain for the development of cutting‐edge technologies across a wide range of fields, including machine learning, scientific computing, similarity search, neural networks, security electronics, and edge computing etc.

The schematic illustration of RC with memristors showcases a system capable of handling a variety of input types, including digital, analog, synaptic, sensory, and masking (pattern) inputs. This versatility enables complex processing tasks for diverse applications such as classification, forecasting, in‐sensor computing, visual perception, edge computing, and bionic vision (in Figure 1a). Inspired by biological visual systems (in Figure 1b), the in‐memory sensor‐based RC system was developed with a configuration that includes a reservoir encoder based on a photo‐synaptic memristive device array [48]. In the human visual perception, the detection and transmission of external stimuli are handled by a network of receptors (such as the retina), neurons, and the visual cortex in the brain. This arrangement allows the system to effectively tackle complex and unstructured real‐world challenges. Visual signals are initially captured by the retina, then transmitted through a network of neurons and synapses, and subsequently processed in the visual cortex, which is responsible for tasks such as memory encoding, learning, and object recognition [49]. This bio‐inspired RC architecture represents a significant departure from conventional sensing methods by enabling simultaneous sensing and processing. This approach not only enhances efficiency but also minimizes power consumption, aligning with the principles of biological systems where parallel processing and adaptive responses are integral. By emulating the brain's ability to handle multiple tasks concurrently, this architecture offers a more robust and energy‐efficient solution for complex sensory and computational tasks [50]. The RC system primarily comprises a reservoir layer and an output readout layer, as depicted in the dashed box of Figure 1c. The reservoir's states in an RC system need to be nonlinearly mapped by temporal inputs and correlate with their previous states. The readout layer's weights are trained for specific learning tasks, and the final outputs typically rely on a linearly weighted sum of the reservoir's feature outputs. The persistence photoconductivity (PPC) effect, which exhibits nonlinearly tunable characteristics, makes WS_2_ photo‐synapses excellent candidates for photoelectronic reservoirs, as illustrated in the left panel of Figure 1c.

Memristors, known for their excellent adjustable resistive state characteristics and their natural ability to perform vector‐matrix multiplication in an array structure, are well‐suited for use as the readout layer [51, 52], as shown in the right panel of Figure 1c. Consequently, we have developed an in‐memory sensor‐based RC reservoir encoder system utilizing a photonic memristive synaptic device. The fabrication process of the photo‐synaptic device is shown in the (Note S1 and Figure S1). Advancements in this domain facilitate the development of futuristic technologies across various fields, including machine learning, scientific computing, hardware neural networks, encryption electronics, and edge computing. By integrating material advancements and leveraging the unique properties of memristors, this RC system represents a significant step toward creating efficient, high‐performance computing frameworks that mimic human cognitive processes (Figure 1d).

**FIGURE 2:**
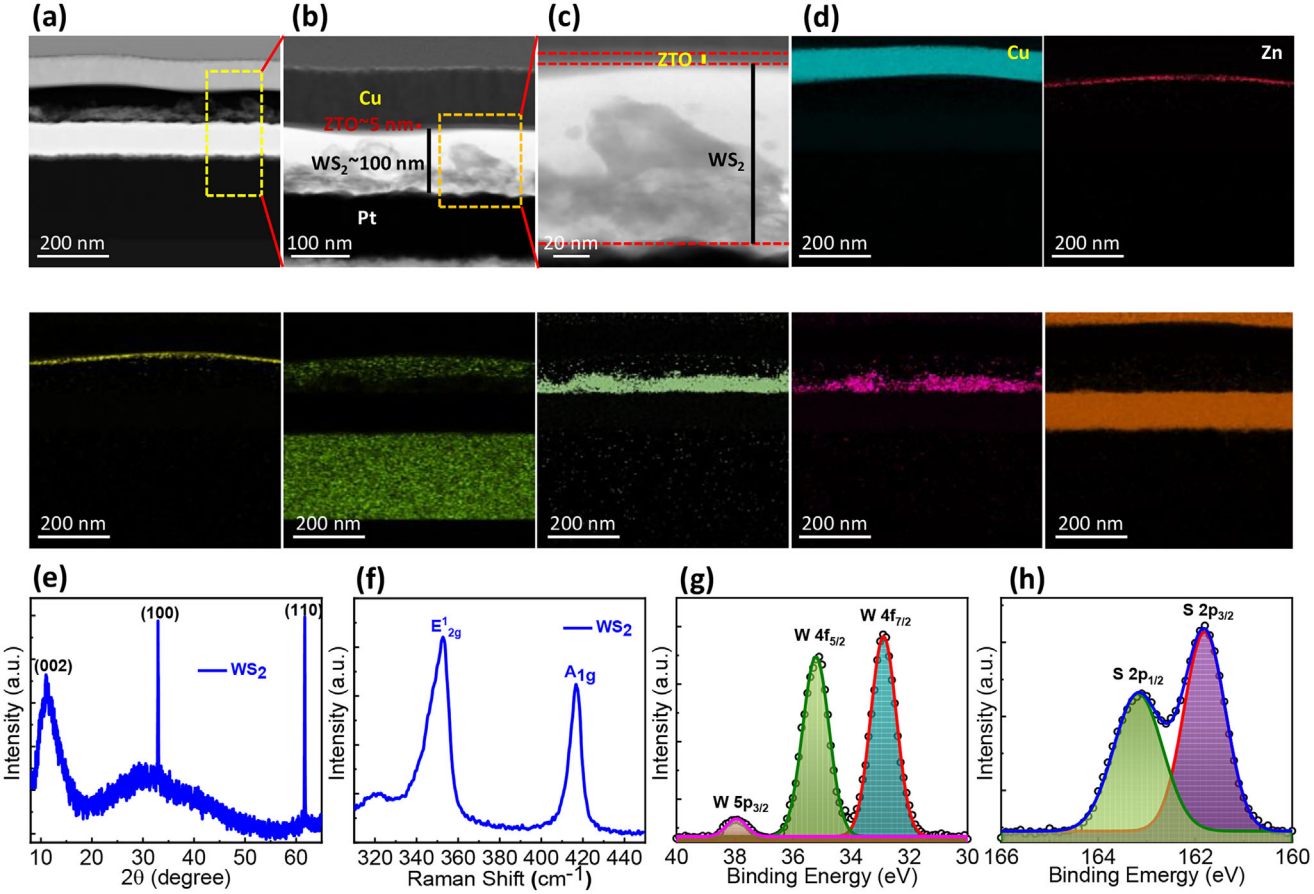
(a–c) High‐resolution cross‐sectional TEM images of the Cu/ZTO/WS_2_/Pt device are presented with scale bars of 200, 100, and 20 nm, respectively. (d) Depicts EDS elemental mapping for Cu, Zn, Sn, O, W, S, and Pt, illustrating their distribution within the device. (e) XRD spectrum of the WS_2_ thin film. (f) Raman spectra of the WS_2_ thin film. (g, h) The depth scan XPS spectra display the W 5p, W 4f, and Zn 2p, peaks, indicating the chemical states within the HfOx and ZnO layers.

To ascertain the precise composition of the fabricated device, comprehensive structural characterizations were conducted, as depicted in Figure 2. High‐resolution transmission electron microscopy (HR‐TEM) images of the Cu/ZTO/WS_2_/Pt device are delineated in Figure 2a–c, with scale bars denoted at 200, 100, and 20 nm, respectively. These images elucidate intricate structural details of the device, showcasing a uniformly 5 nm thick layer of ZTO and an approximately 100 nm thick layer of WS_2_ prominently visible on the Pt bottom electrode. Elemental mapping encompassing Cu, Zn, Sn, O, W, S, and Pt validates the existence of a multilayer structure within the Cu/ZTO/WS_2_/Pt configuration Figure 2d. Furthermore, the X‐ray diffraction (XRD) spectra in Figure 2e exhibit diffraction peaks at various angles, thereby corroborating the presence of WS_2_ within the structure [31, 53, 54]. In Figure 2f, the Raman spectrum exhibits distinct peaks attributed to the *E* ^1^_2g_ and *A*_1g_ phonon modes of WS_2_, observed at frequencies of 352.08 and 418 cm^−1^, respectively. Notably, the mode difference of 65.92 cm^−1^ indicates the presence of multilayered WS_2_ [55, 56, 57]. The XPS results illustrated in Figure 2f,g substantiate the surface chemical composition and chemical valence of WS_2_ elements. The peaks observed at 32.8, 35.4, and 38.1 eV correspond to W 4f_7/2_, W 4f_5/2_, and W 5p_5/2_, respectively (Figure 2g), while the divalent S 2p3/2 and S 2p1/2 peaks of sulfide ions are identified at 161.8 and 163.1 eV, respectively (Figure 2h). These findings confirm the bonding energies of W^4+^ and S^2−^ within WS_2_ [58, 59]. The detailed study of thin film quality control thickness of WS_2_ is shown in (Note S2 and Figure S2).

**FIGURE 3:**
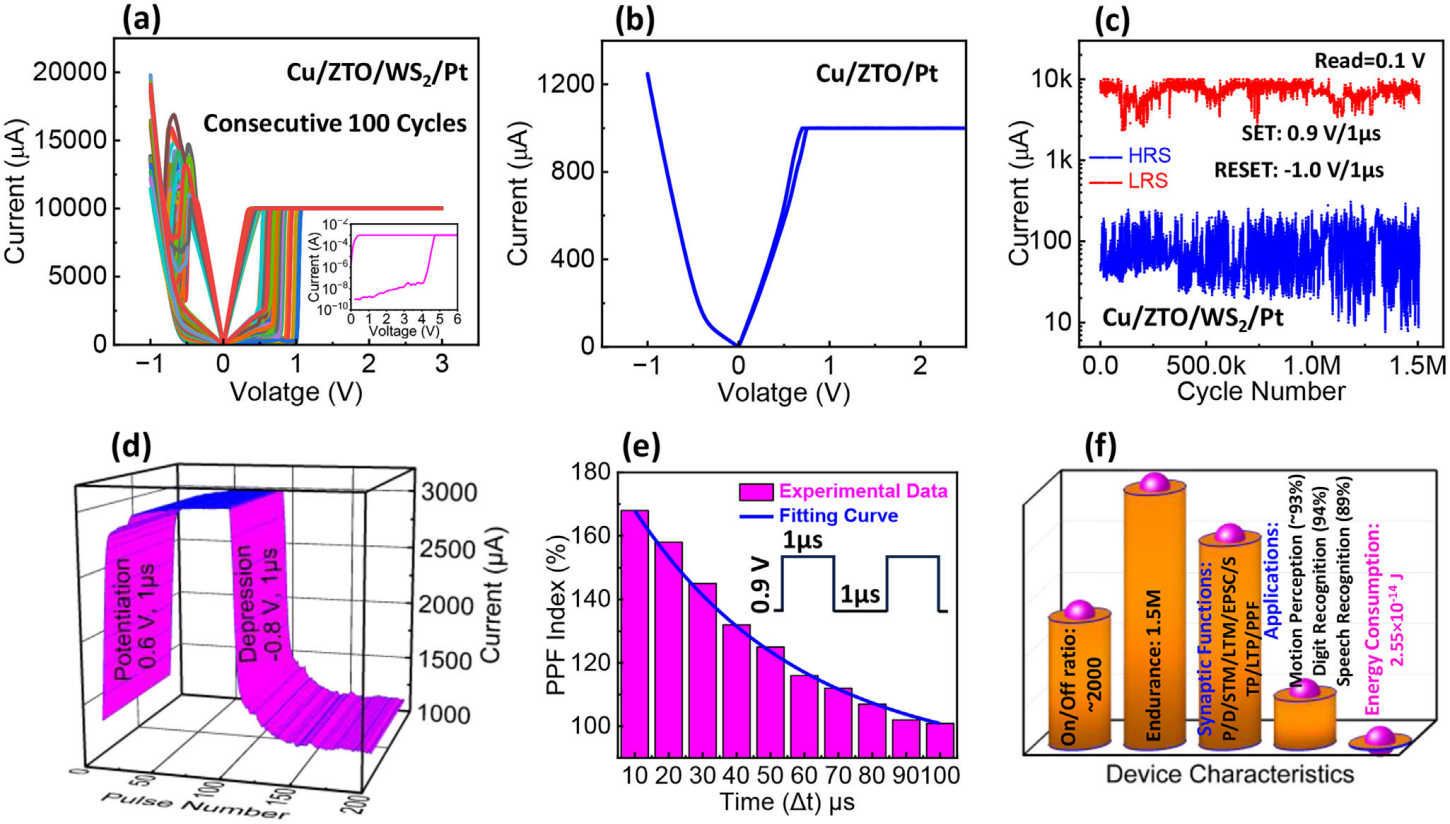
(a) *I–V* characteristic of the 100 consecutive cycles of Cu/ZTO/WS_2_/Pt device. (b) *I–V* characteristics of the Cu/ZTO/Pt device. (c) High stable AC endurance of the device. (d) P and D of the device. (e) PPF index of the device. (f) Device characteristics.

The electrical and synaptic properties of the device were experimentally demonstrated, as depicted in Figure 3. Figure 3a depicts the *I–V* characteristics of the Cu/ZTO/WS_2_/Pt memristor. The device consistently demonstrates stable switching behavior for 100 consecutive cycles during both SET and RESET procedures. The electroforming procedure was performed using a voltage sweep applied to the Cu top electrode in the range of 0–+6.0 V, with a current compliance of 1 mA to prevent hard breakdown. The sweep was conducted with a step size of 10 mV and forming was done at around 4.5 V. Successful forming was defined by the appearance of a sudden current increase accompanied by a stable transition into a low‐resistance state that could be reproducibly switched in subsequent SET/RESET cycles (inset of Figure 3a). To confirm that WS_2_ serves as the primary switching layer in the Cu/ZTO/WS_2_/Pt memristor, we investigated the *I–V* characteristics of a Cu/ZTO/Pt device (Figure 3b). The absence of a characteristic resistive switching memory window in this device provides validation for the crucial role of WS_2_ as the main switching layer in the Cu/ZTO/WS_2_/Pt configuration. The long‐term AC endurance of the device is depicted in Figure 3c. The *I–V* characteristics of the Cu/WS_2_/Pt and Cu/ZTO/Pt devices are shown in the (Note S3 and Figure S3). The highly stable long‐term AC endurance of the device in both Low Resistance State (LRS) and High Resistance State (HRS) confirms the reliability of the device. The endurance of the device was measured at a SET voltage of 0.9 V and a RESET voltage of −1 V with a pulse width of 1 µs in both SET and RESET operations. The read voltage was fixed at 0.1 V during the measurement. The reliability and variability assessments are depicted in (Note S4 and Figures S4–S9). The synaptic weight potentiation (P) and depression (D) features of the device using input voltage pulses are shown in Figure 3d. The voltage pulse scheme of the device is shown in (Note S5 and Figure S10). The conductance of the synapse can be increased or decreased using these identical potentiation and depression pulses.

In a biological neural system, PPF serves as a key synaptic function, showcasing the capability to process sequential temporal information. PPF exemplifies temporal summation at biological synapses, boosting synaptic strength by reducing the time between two consecutive enhancement pulses. To explore how PPF depends on pulse intervals, we tested and determined the PPF index across various intervals, as depicted in Figure 3e. The findings indicate that the PPF index diminishes exponentially as pulse intervals lengthen. Furthermore, the relationship between the PPF index and pulse intervals can be modeled using a double‐exponential function [52, 60, 61]:

(1) $$\mathrm{PPF}=1+C_{1}\exp\;(-\Delta t/\;\tau_{1})+\;C_{2}\exp\;(-\Delta t/\tau_{2})$$

In this model, C_1_ and C_2_ represent the facilitation ranges, and τ_1_ and τ_2_, with fitted values of 4.97 and 45.21 s, respectively, are the characteristic time constants corresponding to rapid and slow relaxation times. Consequently, shorter pulse intervals close to the rapid relaxation constant, τ_1_, show a higher PPF index or facilitation efficiency. This enhances the STP capability, making the outputs generated by different inputs more distinguishable. Figure 3f summarizes the device characteristics, highlighting application accuracies and power consumption.

**FIGURE 4:**
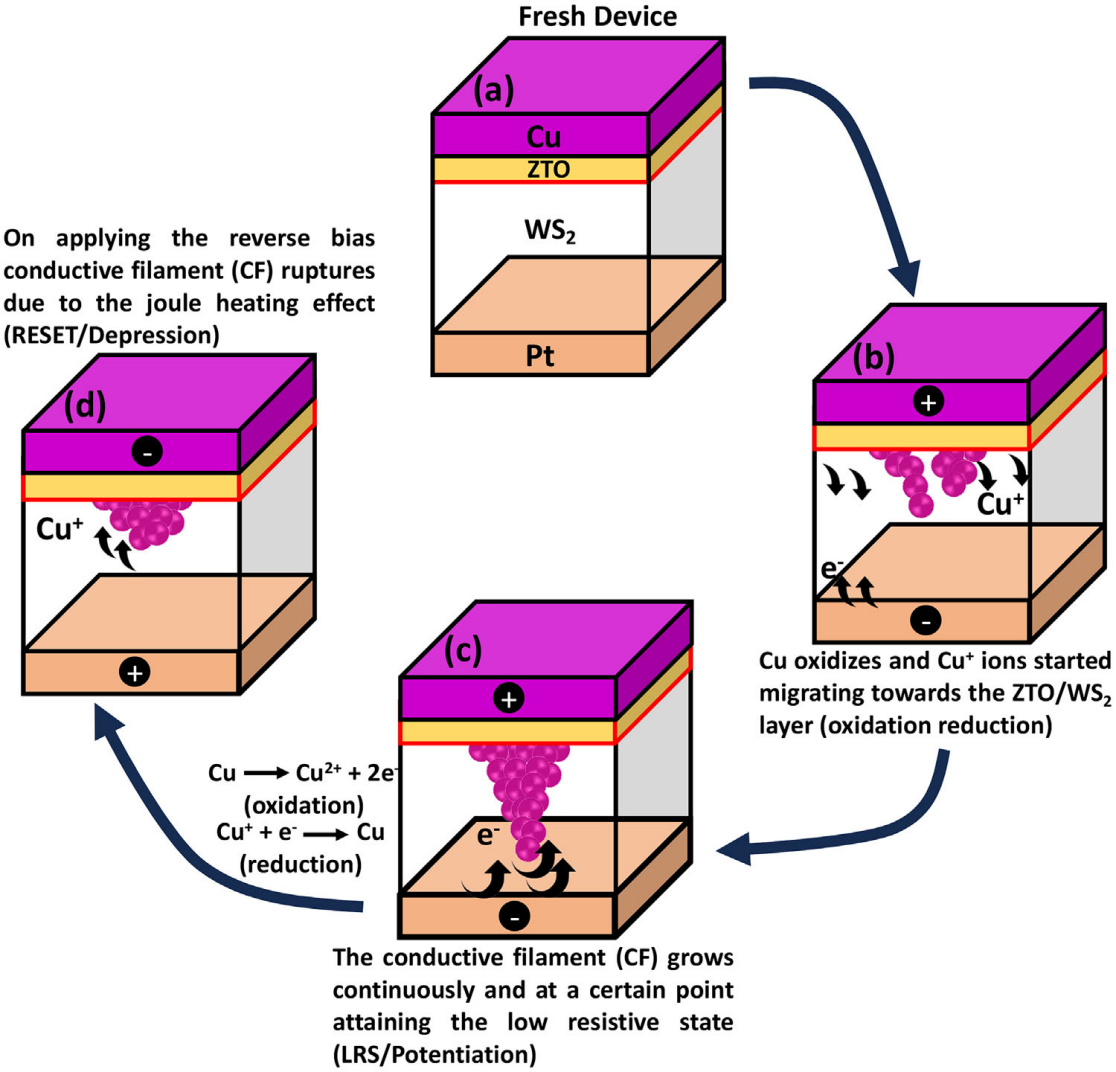
Depicts electrochemical processes involved in resistive switching. (a) pristine state of the Cu/ZTO/WS_2_/Pt device. (b) Initially, Cu atoms are oxidized, releasing Cu^+^ ions into the WS_2_ layer. As Cu^+^ ions migrate through the switching layer, a reduction reaction takes place before these ions reach the Pt. (c) The continued growth of the conductive filament (CF) eventually results in a short circuit between the two electrodes, establishing the low resistive state. (d) Reversing the bias triggers a Joule‐heating‐assisted electrochemical reaction that causes the CF to rupture at its narrowest point, where maximum power dissipation occurs. This process leads to the disconnection of the conductive path, creating a high resistive state.

To enhance comprehension of the results presented, a proposed mechanism delineates the formation and rupture of the conductive filament (CF), which underlies the resistive switching behavior. Initially, Figure 4a depicts the device's original configuration prior to any bias application. Upon applying a positive bias to the top electrode, Cu atoms are oxidized to Cu^+^ ions and begin to migrate into the WS_2_ layer, as illustrated in Figure 4b. As the voltage increases, the oxidation and reduction processes shown in Figure 4c facilitate the complete growth of the CF. This phase, known as the SET or the low resistive state (LRS), signifies full filament development. Conversely, applying a negative bias, as demonstrated in Figure 4d, leads to CF rupture due to Joule heating, transitioning the device to the RESET stage, or the high resistive state (HRS). Although Joule heating plays a crucial role in filament rupture, it is not the sole driving factor, and the RESET operation in our Cu/ZTO/WS_2_/Pt device is governed by a synergistic electro‐thermal and electrochemical process that is inherently polarity dependent. During the SET operation under positive bias, Cu atoms at the active top electrode are oxidized into Cu^+^ ions and drift toward the inert Pt bottom electrode, where they are reduced to form a metallic conductive filament with a narrow and weakly bonded neck region near the inert electrode. When a negative voltage is applied during RESET, two processes occur simultaneously: (i) localized Joule heating, which concentrates at the thinnest filament segment due to current crowding, and (ii) electrochemical dissolution of the filament, where the reversed electric field promotes re‐oxidation of Cu atoms back into Cu^+^ ions and drives them away from the filament tip. This polarity‐induced redox reaction significantly lowers the energy barrier required for filament rupture compared to Joule heating alone, thereby enabling reliable RESET at lower power. In contrast, applying a large positive voltage, although it generates Joule heating, promotes continuous Cu ion injection and filament growth rather than dissolution, resulting in inefficient or unstable RESET operation. Such polarity‐controlled RESET behavior has been extensively reported in electrochemical metallization (ECM) memristors [62, 63, 64, 65, 66], where the rupture process is dictated by the combined effects of Joule heating and reverse‐bias‐driven electrochemical reactions rather than purely thermal breakdown. Therefore, in the device, Joule heating assists filament rupture, but the application of a negative bias is physically essential to reverse the electrochemical driving force, initiate filament dissolution, and ensure stable, repeatable RESET behavior. In filamentary ECM devices, the LRS conduction is governed by the formation of a highly localized metallic Cu filament with a nanoscale diameter, which is significantly smaller than the micrometer‐scale device area. As a result, the LRS current is determined by filament geometry rather than the total device area and therefore exhibits weak or negligible dependence on device size. In contrast, in the HRS, the metallic filament is ruptured, and conduction occurs through defect‐assisted transport across the switching layer, which may exhibit partial area dependence. This difference in area scaling between LRS and HRS is a well‐established characteristic of ECM‐type memristive devices and has been experimentally verified in numerous Cu‐ and Ag‐based filamentary switching systems, where the LRS resistance remains nearly constant across different device sizes while the HRS resistance shows greater variation [67, 68, 69, 70, 71].

## 3 Opto‐Electronic Synaptic Characteristics

**FIGURE 5:**
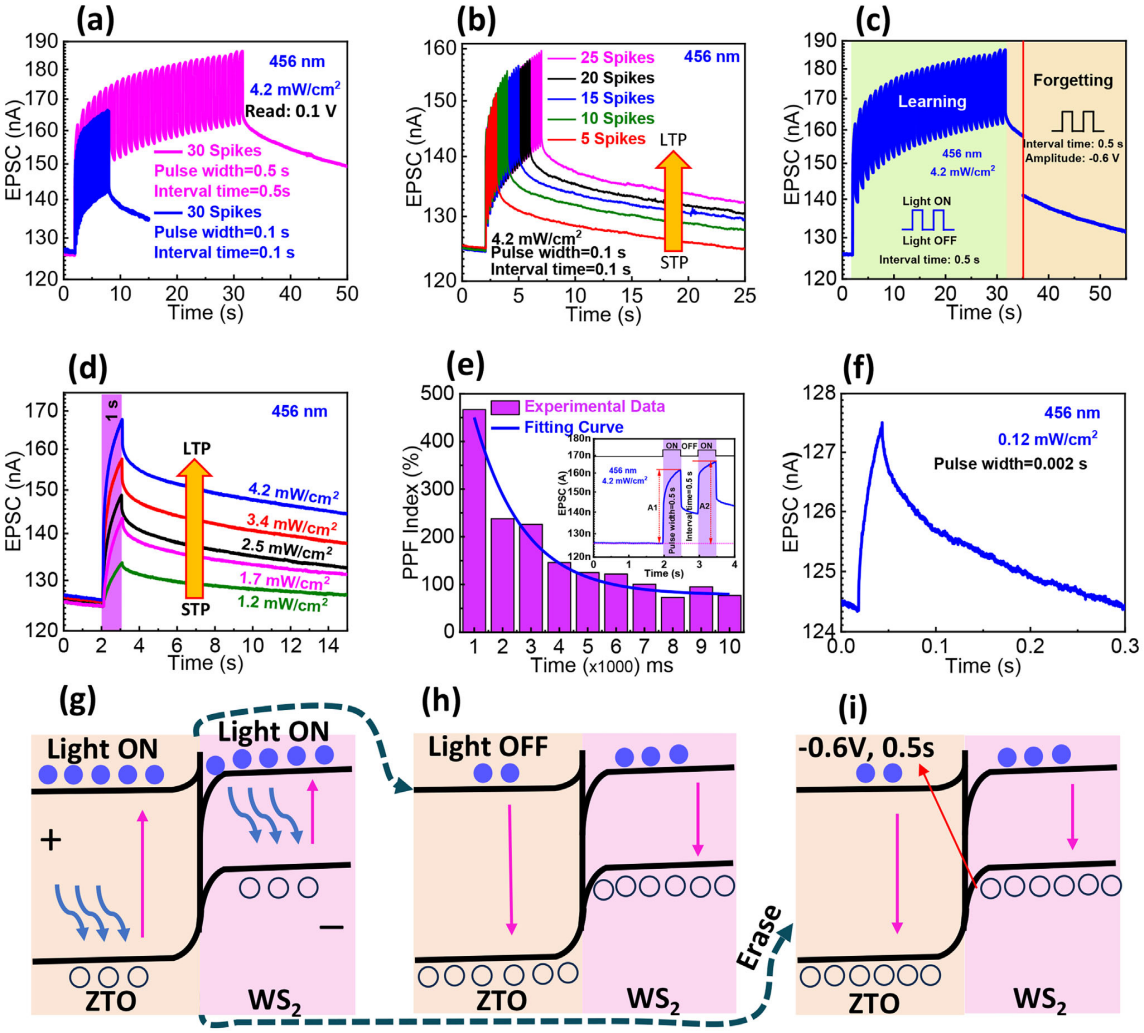
The device characteristics for blue light of 456 nm wavelength (a) EPSC of the device for 30 spikes with pulse width (0.5 s, 0.1 s) and interval time of (0.5 s, 0.1 s) using the 4.2 mW/cm^2^ intensity. (b) EPSC of the device with the pulse width of 0.1 s and interval time of 0.1 s for 5, 10, 15, 20, 25 spikes, respectively. (c) Learning, forgetting, and relearning behaviors under successive optical and electrical pulses. (d) PPF index variation with time interval (in inset: EPSC under the wavelength of 456 nm pulses pair with the time interval of 0.5 s). (e) EPSC of the device for the short pulse width of 0.002 s with small intensity of 0.18 mW/cm^2^. (f) Energy consumption of our device with reported one. The energy band alignment of the ZTO/WS_2_ heterojunction (g) under the illumination of light (h) after turning off the optical light, (i) under the negative voltage pulse (−0.6 V, 0.5 s).

STP plays a key role in recognizing and processing transient input information, while LTP is linked to the formation of enduring learning and memory, which can last several hours or more in the human brain. The transition from STP to LTP can be induced by amplifying factors such as stimulation intensity, duration, and pulses, which consequently increase the synaptic weight. In the context of a synaptic memristor, this transition from STP to LTP can be accomplished by augmenting parameters like intensity of light, spike duration, and spike numbers [1, 12, 72, 73, 74]. Figure 5a shows the EPSC triggered by an optical spike as a function of the spike duration under the same light intensity of 4.2 mW/cm^2^. The results confirm that the EPSC increases with increasing pulse duration. Figure 5b depicts the EPSC with varying spike numbers with intensity of 4.2 mW/cm^2^. A short spike triggers a small EPSC that quickly decays, whereas a longer spike initiates a larger EPSC that decays at a much slower rate, allowing the response to be retained for a longer time, depicting the transition from STP to LTP. The branching point between STP and LTP is shown in (Note S6 and Figures S11 and S12) and the other synaptic characteristics of the devices prior to the electroforming are shown in (Note S7, Figures S13–S15).

In human cognition, memory enhancement typically requires repeated learning, which can decelerate the rate of forgetting. Based on the LTP, we further examined the learning and memory capabilities of the device (Figure 5c). The intriguing observation is that the device demonstrates enhanced LTP characteristics when subjected to optical pulse stimuli, while exhibiting a tendency toward forgetting when exposed to electrical pulses, suggesting a light‐potentiation and electrical erase behavior. The EPSC of the device was modulated by applying optical stimuli with different intensities (1.2, 1.7, 2.5, 3.4, and 4.2 mW cm^−^^2^), as shown in Figure 5d. Higher light intensity results in a larger increase in conductance and affects the decay dynamics of the photocurrent. At lower light intensities, the EPSC exhibits only a slight increase and rapidly returns to its initial baseline after the light is turned off, mimicking STP behavior in the human brain. In contrast, under higher light intensities, the PSC increases significantly and decays more slowly after stimulus removal, remaining at an elevated level above the initial state, which resembles LTP characteristics in biological synapses. We carried out further investigations into the light‐irradiated synaptic plasticity PPF of the device, as shown in Figure 5e. The investigation of photonic PPF was conducted in a similar manner to that of electrical PPF, as described by Equation (1). The resultant observations excellently mimic the synaptic photonic PPF characteristic, which holds significant value as a synaptic function within the framework of neuromorphic vision systems. Figure 5f depicts the EPSC of the device for the short pulse width of 0.002 s with small intensity of 0.12 mW/cm^2^ and the energy consumption per event of the optical device was ∼2.5 × 10^−^^14^ J. The optical energy consumption per spike is calculated using the relation *E*  =  *I* × *V* × *t*, where *I* is the measured device current under illumination, *V* is the applied read voltage during synaptic operation, and *t* is the optical pulse duration [75, 76]. The optical synaptic characteristics of the devices for 532 nm wavelength are shown in the (Note S8 and Figure S16).

As photoelectric detection materials, both ZTO and WS_2_ exhibit strong photocurrent responses owing to their intrinsic photoconductive properties. In our device, the observed optoelectronic synaptic behaviors arise primarily from the synergistic photoconductive effect of the ZTO/WS_2_ heterostructure. As illustrated in Figure 5g, upon light illumination, the incident photons generate electron–hole pairs; the electrons are excited from the valence band to the conduction band, leaving behind holes in the valence band. This carrier generation and separation process enhances the carrier density in the channel, thereby increasing the current and producing a pronounced light‐induced conductance increase [77, 78, 79]. Once the illumination is turned off, the device exhibits a characteristic decay in photocurrent, which is attributed to persistent photoconductivity. Specifically, the photoexcited electrons gradually recombine with the remaining holes in the valence band, reducing the number of free electrons in the conduction band and leading to a slow relaxation and. decay of the current, as depicted in Figure 5h. During the electrical erase process, the application of a negative voltage pulse to the top Cu electrode forces trapped charges to de‐trap and recombines with hole cousin erasing effect via electrical pulse. This results in a sharp reduction in conductance. Thus, after the light is turned off, the photo‐generated excitons recombine with electrons through direct tunneling under the applied negative voltage [77]. This recombination mechanism sharp decreases the conductance, as shown in Figure 5i.

It is important to distinguish between the optoelectronic synaptic erase process and the electrical RESET process in the Cu/ZTO/WS_2_/Pt device, as they operate under different physical regimes. In the optoelectronic synaptic mode, the enhanced conductance after light stimulation originates primarily from photogenerated carriers and trap‐assisted transport in WS_2_ and at the ZTO/WS_2_ interface, rather than from a fully developed metallic Cu conductive filament. Persistent photoconductivity in WS_2_ is well known to arise from defect‐mediated carrier trapping and interfacial band modulation, which can produce long‐lived conductance states without requiring metallic filament formation [80, 81, 82]. When a negative voltage pulse is applied in this photo‐programmed state, the electric field accelerates electron de‐trapping from defect states and promotes recombination with holes via field‐assisted tunneling, while simultaneously restoring the interfacial barrier height. This process leads to a rapid decay of the photo‐induced conductance and constitutes the synaptic erase operation shown in Figure 5i. In this regime, the conductance modulation is dominated by electronic relaxation and trap‐mediated carrier recombination processes, which are characteristic of optoelectronic synaptic devices based on 2D semiconductors [82, 83]. During electrical resistive switching, the device operates in a classical ECM mode. A positive bias on the Cu top electrode induces oxidation of Cu atoms into mobile Cu^+^ ions, which migrate through the switching layer and are reduced near the inert electrode to form a metallic conductive filament, establishing the low‐resistance state [67, 69]. A reversed bias triggers filament rupture through electrochemical dissolution, assisted by localized Joule heating at the filament constriction, resulting in an abrupt transition from the low‐resistance state to the high‐resistance state [67, 69, 70]. In this electrically formed filamentary regime, Cu filament rupture is the dominant mechanism governing resistance switching. Therefore, the negative voltage pulse plays distinct roles depending on the initial device state: it drives charge de‐trapping and conductance relaxation in the optoelectronic synaptic regime, and it induces Cu filament rupture in the electrical RESET regime. This clear separation of operational modes reconciles the observed conductance modulation mechanisms and ensures a consistent interpretation of both the optoelectronic synaptic behavior and the electrical resistive switching characteristics.

We have validated the mechanisms by first principles calculations using quantum EXPRESSO in the (Note S9 and Figure S17).

## 4 Device Testing in Humid Conditions

**FIGURE 6:**
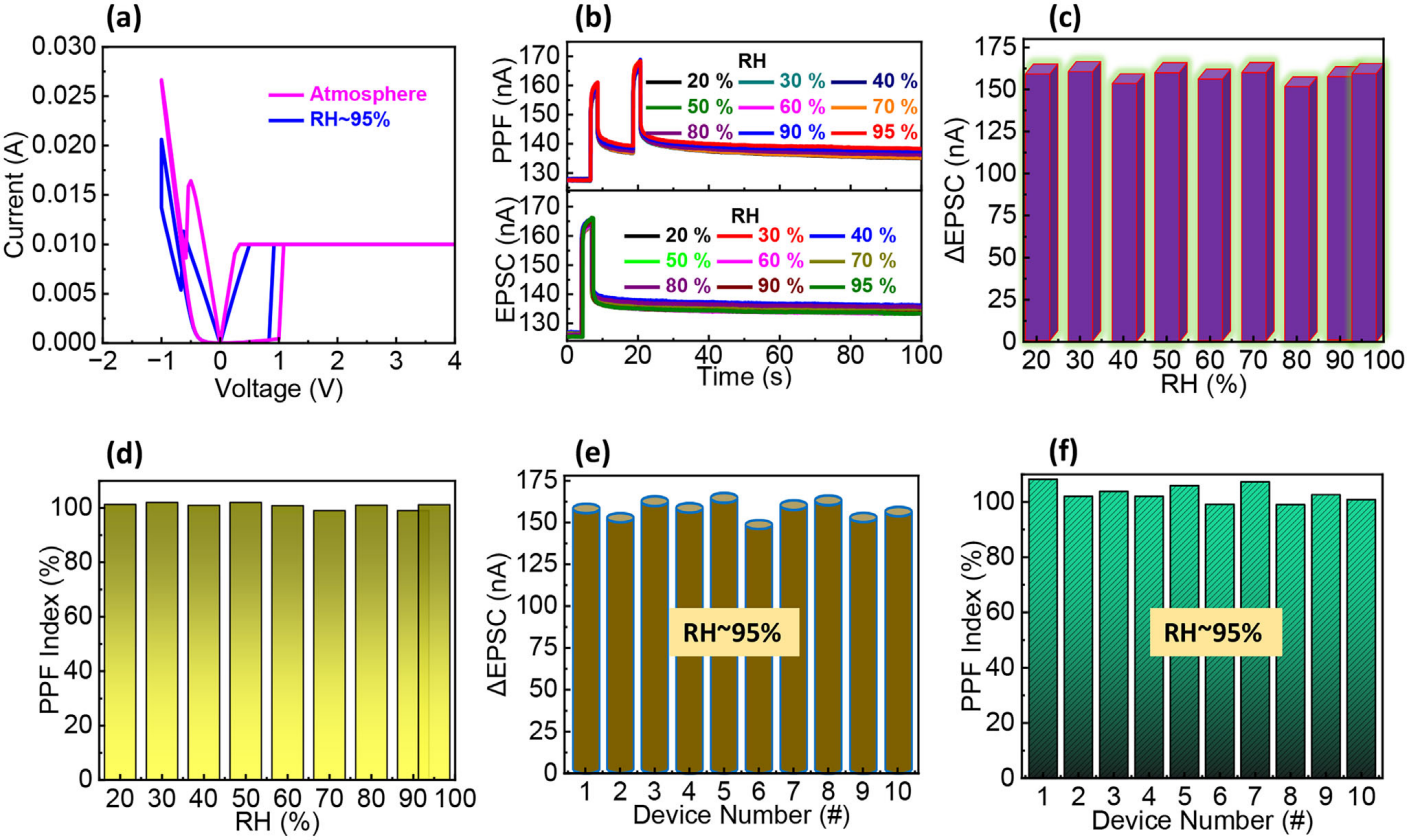
(a) *I–V* characteristics of the device in air and relative humidity (RH) of 95%. (b) EPSC and PPF features of the device at various levels of humidity. (c) EPSC with various levels of humidity. (d) PPF ratio with different relative humidity levels. (e) EPSC for 10 randomly chosen devices at 95% humidity. (f) PPF ratio for 10 devices at 95% of humidity.

To evaluate the environmental resilience of the Cu/ZTO/WS_2_/Pt device, we analyzed its EPSC and PPF responses under various humid conditions. The device's synaptic function shows minimal disruption from water molecules. This stability is likely due to the top ZTO layer acting as an encapsulant, in addition to the W and S molecular radicals’ robust electron‐trapping capabilities, which successfully neutralize the impact of external water on charge trapping [84]. To thoroughly investigate the device's remarkable tolerance to water molecules, we conducted an extensive study on the synaptic behaviors at various RH levels (20%–95%). Figure 6a represents the I–V characteristics of the device in an atmosphere and 95% humid condition. Figure 6b illustrates the typical EPSC and PPF characteristics, which were analyzed and compared. The PPF behavior, which reflects the ratio of two consecutive postsynaptic current levels, is a well‐known short‐term memory effect that underlies human memory processes. Our results showed that despite the high RH, our device maintained typical EPSC and PPF behaviors, highlighting its robustness in mimicking these memory‐related phenomena. Figure 6c,d presents the derived EPSC values and PPF ratios at various levels of RH (20%–95%), showing relatively stable performance. The device‐to‐device derived EPSC and PPF behaviors were also measured at high humid conditions (95%) for randomly chosen 10 devices from the sample (Figure 6e,f). All devices exhibited minimal variation in derived EPSC and PPF behaviors, signifying a remarkably stable performance. These observations imply the absence of an additional charge‐trapping phenomenon by water on the WS_2_ layer, leading to negligible disruptions in synaptic functionality under different humidity levels. The sputtered or ALD‐deposited amorphous Zn─Sn─O films have been reported to exhibit low WVTR values in the range of ∼10^−^^4^–10^−^^6^ g·m^−^^2^·day^−^^1^, depending on thickness, density, and deposition conditions, which is comparable to well‐established oxide encapsulation materials such as Al_2_O_3_ and SiN_x_ [85, 86, 87, 88, 89]. These literature reports attribute the low WVTR to the dense amorphous microstructure and strong Zn─O and Sn─O bonding network, which effectively suppress water‐vapor diffusion [86, 90, 91]. We now explicitly cite these references in the revised manuscript wherever WVTR‐related claims are made. Furthermore, we clarify that the observed humidity robustness of the Cu/ZTO/WS_2_/Pt device is attributed primarily to the encapsulation of the ZTO layer, with additional contribution from the intrinsic surface chemistry of WS_2_. The ZTO layer acts as a physical moisture barrier that limits water‐vapor penetration to the underlying WS_2_ surface, thereby preventing moisture‐induced charge trapping and interfacial instability. At the same time, WS_2_ is known to exhibit relatively stable surface chemistry compared to many oxide semiconductors, with defect‐mediated trapping states that remain functionally stable under moderate moisture exposure [92]. Therefore, the excellent synaptic stability observed under high humidity (up to 95% RH) is primarily governed by the protective encapsulation effect of the ZTO interlayer, while the intrinsic defect‐tolerant electronic properties of WS_2_ further support stable optoelectronic operation.

Consequently, these outcomes underscore the device's resilience in high‐humidity conditions, indicating that external moisture does not significantly impact its performance. The humidity testing setup and PPF ratio with time at 95% humid condition are described in the (Note S10 and Figure S18).

## 5 In‐Memory Sensing, Memorizing, and Visual Perception Systems

**FIGURE 7:**
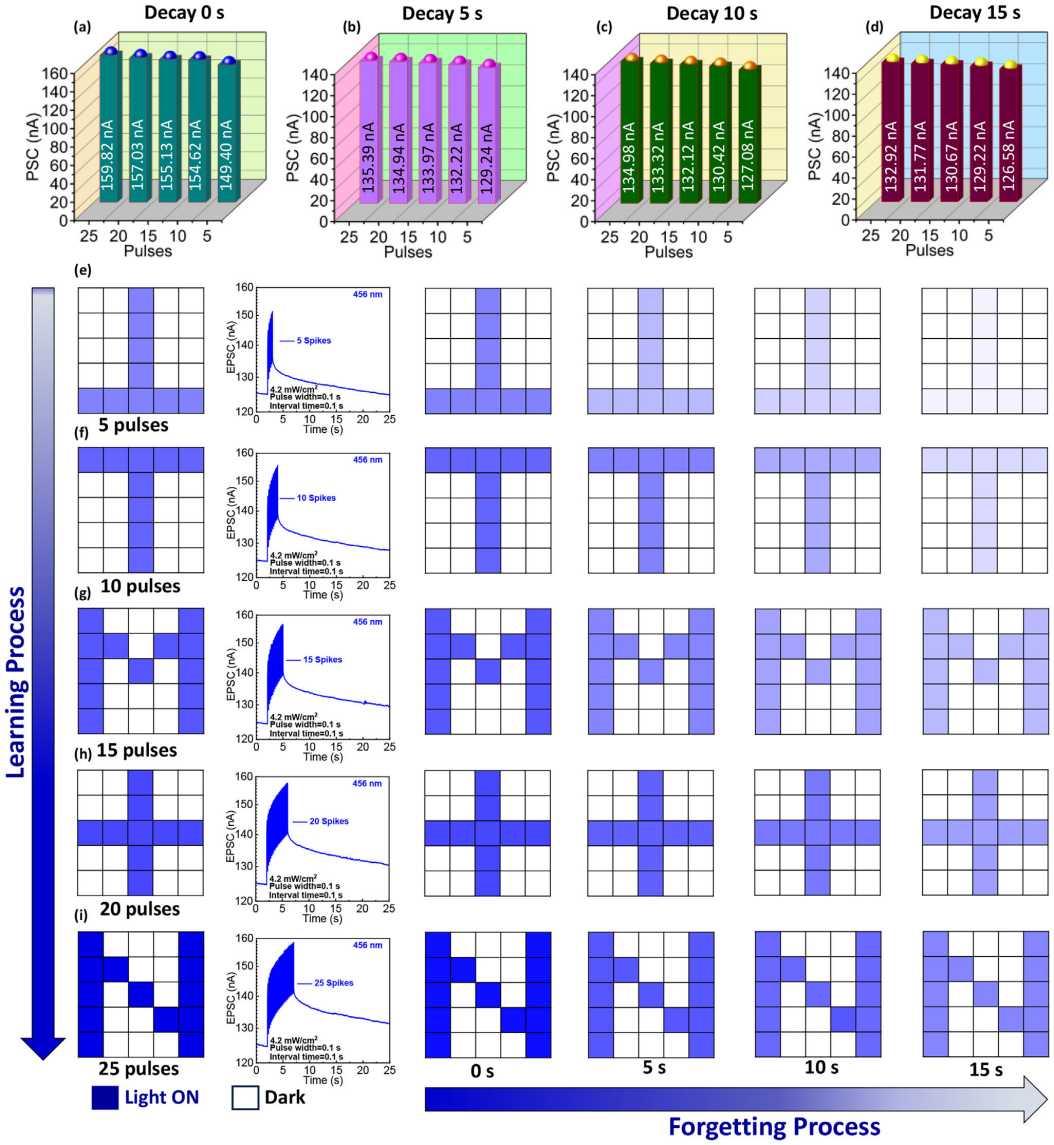
The current conductance of the optoelectronic memristor after various numbers of pulses at (a) 0 s, (b) 5 s, (c) 10 s, and (d) 15 s decay time. The image mapping for current conductance after (e) 5 pulses, (f) 10 pulses, (g) 15 pulses, (h) 20 pulses, and (i) 25 pulses.

Studies on visual memory suggest that retention can be enhanced either by increasing the number of pulses, a process referred to as memory recall. In this work, we investigated the visual memory performance of a 5 × 5 device array operating under a “learning, forgetting, and relearning” scheme. Mimicking the human visual system, the array can detect optical vision pulses and induce corresponding changes in PSC. Figure 7a presents the photocurrent responses for different pulse counts (5, 10, 15, 20, 25), showing a clear increase in PSC with more pulses, including a 10.42 nA difference between the 5th and 25th pulses. Figure 7b–d depicts the conductance states for various pulse stimuli at decay times of 5, 10, and 15 s, demonstrating that higher pulse numbers strengthen memory retention and extend the forgetting period. To further examine the visual sensing capability, we projected the characters “⊥,” “T,” “M,” “+,” and “N” using 5, 10, 15, 20, and 25 pulses, respectively (Figure 7e–i). The character “⊥” faded rapidly and became indistinct, indicating that a single cycle is insufficient for sustained image retention. In contrast, the character “N” remained clearly visible even after a 15 s decay with 25 pulses. By modulating the light stimulus, we were able to control the device conductance in accordance with the “learning, forgetting, and relearning” process, without the need for additional voltage pulses, effectively replicating human visual perception. These results validate the array's ability to learn, retain, and recall visual patterns and highlight its promise for future artificial visual perception systems in electronic vision applications.

## 6 Nonlinear Mapping of 5‐Bit Inputs of the WS_2_ Based Reservoir

**FIGURE 8:**
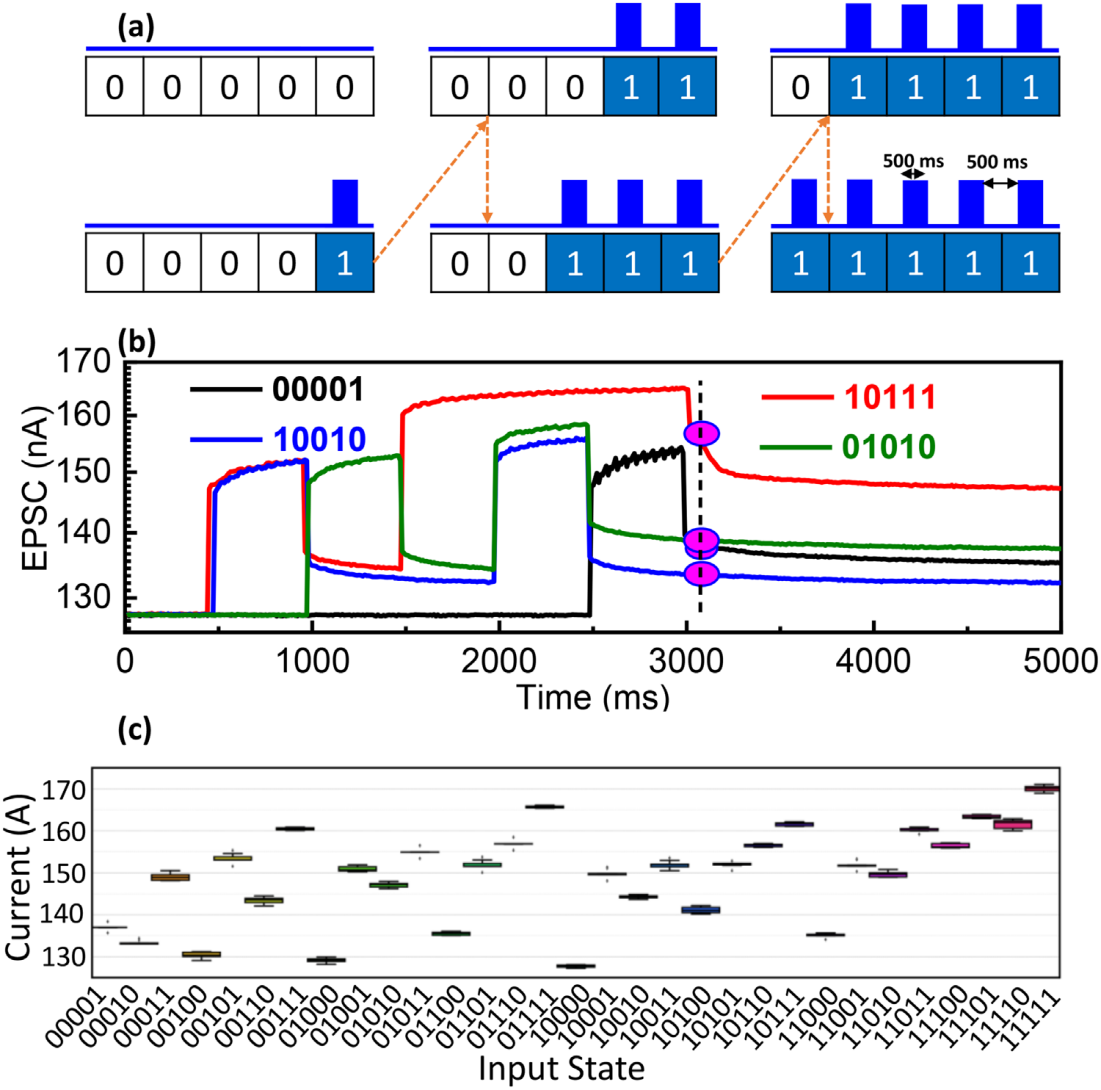
(a) 5‐bit inputs in the form of a binarized number and equivalent light pulses from "00000" to "11111". The pulse width and interval are 500 ms. (b) *I–t* photo response characteristics and input–output feature extraction strategy of four representative inputs of "00001", "10010", "10111", and "01010". (c) Experimental shows the current behavior under thirty‐two 5‐bit optical pulse ranging from (00000) to (11111). Each data point is taken from 10 random chosen devices. For each input state contains five pulses, '1' denotes an optical pulse with a light intensity of 4.2 mW/cm^2^ and a pulse width of 0.5 s while '0' denotes a no‐light situation with a pulse width of 0.5 s.

The process of feature extraction from the original image streamlines recognition tasks and improves efficiency, as highlighted in the referenced literature [1, 51]. The nonlinear PPF of synaptic plasticity allows for the distinct mapping of input sequences in the WS_2_‐based photo‐synapse reservoir into feature outputs. To evaluate this feature mapping capability, we conducted measurements using serials of optical stream, which corresponds to 5‐bit inputs ranging from “00000” to “11111” as illustrated in Figure 8a. As shown in Figure 8a each input waveform consists of a 500 ms pulse width and a 500 ms interval per bit, where the ON and OFF states of the light pulses correspond to binary values “1” and “0”, respectively. This sequence forms the 5‐bit input representation, where each bit's temporal evolution influences the reservoir state. We then measured the current–time (I–t) characteristics for all possible 5‐bit input sequences. Feature values were derived from both the state currents with the decay characteristics after the full sequence. To demonstrate this feature sampling, *I–t* curves for four representative inputs — “00001” in black, “10010” in blue, “01010” in olive, and “10111” in red — of the WS_2_ reservoir are shown in Figure 8b. Notably, even for identical final pulses as in “11111”, their decay profiles vary after the input sequences. Therefore, the reservoir's final state is influenced not only by the last input but also by its ongoing real‐time state, highlighting the lateral connections within this WS_2_ reservoir [15, 51]. Each pixel sequence is characterized by current sampling, enabling feature extraction of both temporal and spatial information. For precise feature extraction, the sampling point is marked by a black dotted line with purple spheres, defined as the average current value following the decay from the last pulse. Thirty‐two representative optical pulses produce thirty‐two distinctly distinguishable states, as shown in Figure 8c. These pulse trains, ranging from (00000) to (11111), generate clearly separated states, demonstrating a strong ability to map complex spatiotemporal signals into reservoir states, which is essential for multi‐task learning and reservoir event driven encoding [93].

## 7 Multi‐Tasking Validation of the WS_2_ Reservoir Kernel

**FIGURE 9:**
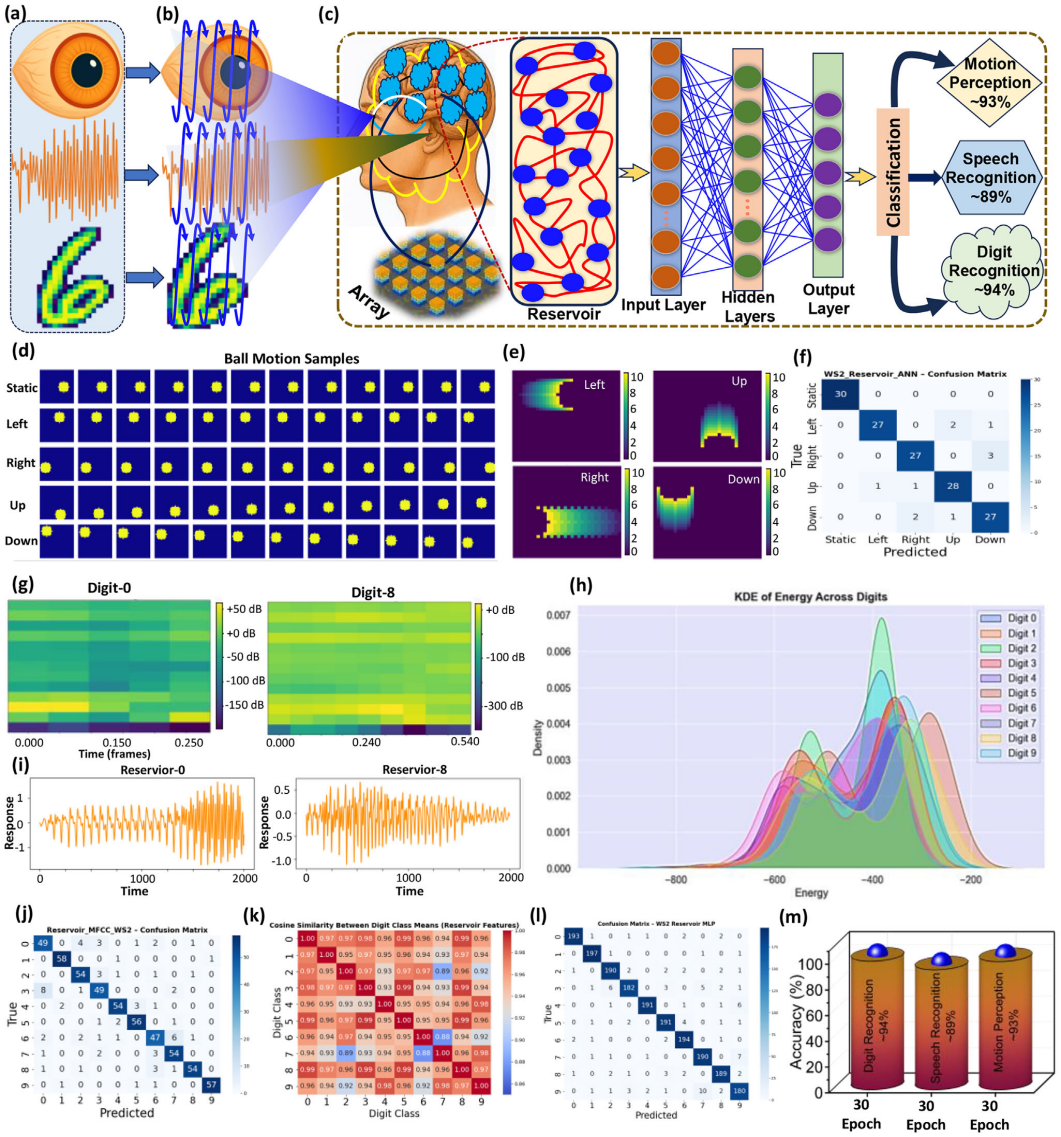
(a–c) Schematic of the unified WS_2_ reservoir computing pipeline, with spike‐based light sequence encoding, non‐linear temporal reservoir processing, and MLP‐based multi‐domain classification. (d) Synthetic ball motion dataset showcasing five classes: Static, Left, Right, Up, and Down. (e) Per‐class energy feature maps extracted from the WS_2_ reservoir responses to synthetic ball motion sequences. For each class (except for static), the total reservoir energy is visualized per pixel after applying the double decay exponential kernel to spike‐encoded input frames. Higher values indicate a region of intense temporal activity, revealing direction‐specific temporal traces. (f) The confusion matrix indicates high classification on the 20% test dataset for each motion direction. (g) MFCC spectrograms for Digit‐0 and Digit‐8 from the FSDD. (h) KDE plot comparing reservoir energy distribution across digit classes, revealing class‐specific patterns in the FSDD. (i) Temporal reservoir response for digit '0' and digit '8' corresponding to Figure 8g, obtained by convolving the spike‐like signal with the kernel. The x‐axis represents time in samples. (j) Confusion matrix for FSDD classification using hybrid MFCC+ WS_2_ reservoir features on 20% test dataset. (k) For the N‐MNIST dataset, a cosine similarity heatmap between reservoir feature class means is represented, showing inter‐class separability. (l) Confusion matrix for N‐MNIST classification using the unified framework. (m) Accuracy comparison across the three tasks: Ball motion perception (∼93%), Speech Recognition (∼89%), and neuromorphic digit recognition with only 30 epochs.

To validate the generalizability of the WS_2_‐inspired reservoir kernel, three distinct pipelines were implemented across multi‐domain sensory modalities within a unified WS_2_‐reservoir kernel computing framework (Figure 9a). The uniform pipeline leverages the experimentally validated synaptic dynamics of the WS_2_ device (Figure 5b), where the 25‐spike regime highlights the STP–LTP transition and establishes stable fading memory. This operating point was selected because the persistent conductance evolution provides durable state retention beyond the transient decay of STP, which is essential for encoding extended sequences. The reservoir kernel is therefore modeled as a double‐exponential decay, directly fitted to the device dynamics (measured EPSC decay curves for single device in Figure 5b using a nonlinear regression approach) details are provided in (Note S11 and Figure S19), with fast and slow relaxation time constants. The extracted parameters were directly implemented in the reservoir model. These values are explicitly implemented in all reservoir simulations (i.e., the kernel is fixed for all tasks). No task‐specific tuning or optimization was performed. This ensures that the demonstrated performance originates from intrinsic device temporal dynamics. Such multi‐timescale fading memory ensures robust temporal encoding using device derived generalized kernel, improving feature separation and classification accuracy across spatiotemporal datasets.

The overall processing flow is shown in Figure 9a–c: raw signals (Figure 9a) are first converted into spike‐encoded representation (Figure 9b) and subsequently passed through the nonlinear fading‐memory kernel extracted from the WS_2_ dynamics (Figure 9c). For motion perception, a synthetic eyeball motion dataset was constructed to simulate controlled 2D spatiotemporal patterns in five directions (Figure 9d). After reservoir processing, each direction produced a distinct energy‐distribution signature (Figure 9e), enabling reliable identification of motion direction. The confusion matrix (Figure 9f) indicates a classification accuracy of ∼93%. Second, for the Free‐Spoken Digit Dataset (FSDD), reservoir dynamics were integrated with MFCC‐based audio features (Figure 9g) were converted into spike‐temporal patterns. The WS2 reservoir generated class‐dependent energy distributions (Figure 9h) and distinct temporal traces (Figure 9i), with ∼89% classification performance while enhancing feature discriminability, demonstrating compatibility with frequency‐domain audio signals. Lastly, the N‐MNIST [94] spike dataset was employed to emulate asynchronous event‐driven vision processing (Figure 9j). The similar matrix of mean reservoir features (Figure 9k) demonstrates strong inter‐class separation, and the resulting confusion matrix (Figure 9l) demonstrates robust performance (∼94%). A consolidated comparison (Figure 9m) highlights that the WS2 kernel effectively supports these different modalities. Thus, across all three cases, the same unified pipeline was applied: raw inputs were first encoded into spike trains to preserve temporal dynamics and mimic neural firing, with motion data converted from inter‐frame differences, FSDD waveforms reshaped into temporal spike‐like signals, and N‐MNIST reconstructed directly from AER format. Each spike train was then processed using a WS_2_‐inspired double exponential decay kernel, derived from empirical device response with fixed parameters (A_1_ = 4.94e‐9, A_2_ = 1.74e‐8, τ_1_ = 1.0749 s, τ_2_ = 44.03 s, y_0_ = 1.19e‐7), introducing fading memory and nonlinearity via convolution. Reservoir responses were subsequently transformed into compact low‐dimensional descriptors, including spatial‐temporal features and additional statistical measures such as entropy and skewness for audio. Finally, classification was performed using a consistent MLP‐based architecture (hidden layers 128 and 64) with an 80/20 train‐test split and 30 training epochs, ensuring methodological uniformity across modalities. To assess statistical robustness, the simulation was repeated across five independent random seeds (as shown in Table S2). This unified technical backbone allows the same WS_2_‐inspired reservoir architecture to be validated across motion sensing, auditory, and neuromorphic visual domains, showcasing the domain‐agnostic nature of the selected reservoir kernel. Additional description of multi‐tasking features is described in Note S11 and Figures S20–S24). In the next section, the event‐driven spike trains are processed through the reservoir mapping demonstrated in Section 6, confirming that the device's intrinsic temporal encoding capability extends beyond simple binary sequences to real spatiotemporal data.

## 8 Opto‐Electronic In‐Memory Sensor‐Based Reservoir Encoder

**FIGURE 10:**
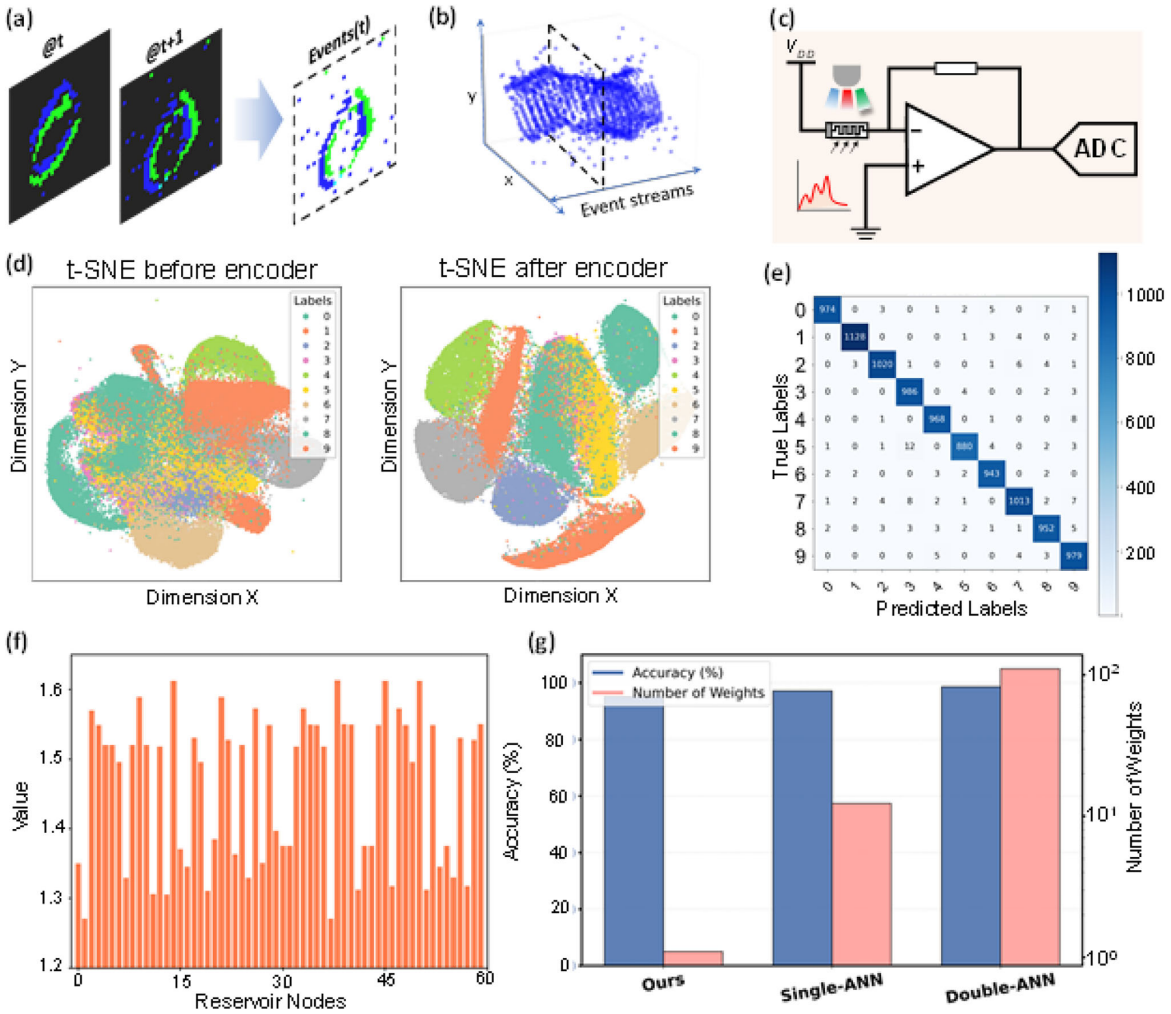
(a) Schematic representation of event‐driven images, where hand‐written digits are represented by positive and negative events. (b) Spatiotemporal view of the spike‐driven event. (c) The proposed circuit diagram, where the WS_2_‐based optoelectronic device works as the reservoir encoder. (d) Distribution of spike samples before and after our reservoir encoder. (e) Confusion matrix visualization of the training result. (f) Randomly selected 60 reservoir nodes and their corresponding values. (g) Comparison of the training accuracy and number of weights between the proposed reservoir‐computing system, a single‐layered ANN, and a double‐layered ANN.

The optoelectronic in‐memory sensor reservoir is illustrated in Figure 10a. Optical stimuli represent the data input into the RC system, which directly mimics the dynamic reservoir behavior with temporal information of device states. Each reservoir node is implemented with a WS_2_‐based device, which independently relies on the physical property of our device. The inherent light‐sensing ability introduces physical non‐linearity with ultra‐low sensing energy consumption and a training‐free framework. The in‐memory sensor reservoir could be effectively used as the photoreceptor and spike encoder, which can help extract the features and maintain the unique advantage of optoelectronic RC over conventional neuron networks. Experimental tests on the proposed in‐memory sensor RC system utilize the temporal optical fading memory for RC and electrical memristive properties for in‐memory computing. First, we evaluate the performance of the reservoir encoder on neuromorphic datasets N‐MNIST [94] (see the details dataset in Notes S12, S25, and S26), which consists of spike‐timing data captured from neuromorphic sensors or DVS camera [95]. We visualize a single data frame of the N‐MNIST dataset with a shape of 34 × 34, as illustrated by the event streams for the digit “0” in Figure 10a and the spatiotemporal view in Figure 10b. The reservoir encoder maps the input spike stream to the spike trains according to the illumination intensity change of the corresponding DVS dataset. Figure 10c illustrates the circuit diagram of the WS_2_ reservoir encoder. Sequences of optical pulses are first perceived by our WS_2_‐based optoelectronic device and then extracted the visual features after the dynamic reservoir, acting as the human retina. The light‐responsive feature can be naturally extracted by the proposed RC system and fed into the electrical memristor crossbar as the readout layer. Figure 10d presents the distribution of spiking events data of test samples with the t‐distributed stochastic neighbor embedding (t‐SNE) method, which demonstrates the data distribution and discriminability in a low dimension. After the WS_2_‐based reservoir encoder, it is noticeable that features become more distinguishable and separable, which helps improve data interpretability in neuromorphic computing scenarios. Figure 10e shows the confusion matrix after the training process, where diagonal elements dominate each row, indicating the high accuracy in the sub‐classification task. Figure 10f visualizes the value of 60 randomly selected nodes in the reservoir encoder layer. Spike behaviors are accumulated and counted with a linear classification head to output prediction results. Figure 10g compares the accuracy and weight trade‐off with single‐layer artificial neural network (ANN) and double‐layer ANN. With a comparable accuracy of 95.1%, our reservoir system holds an affordable training weight of ∼1.1k while that of the other two networks are ∼10.4k and 127k, respectively. Thus, Section 7 validated WS_2_ reservoir kernel generalization, while Section 8 validates device‐level mapping at system scale.

## 9 Conclusion

In conclusion, we have demonstrated the first ultra‐low‐power and humidity‐resilient in‐memory sensor reservoir computing (RC) system using WS_2_ optoelectronic memristors for multitask learning and event‐driven data encoding. This dynamic WS_2_‐based platform uniquely integrates sensing, memory, and computation within a single compact device, enabling the emulation of multiple biological functions. The system achieves strong performance across vision, tracking, and speech recognition tasks with exceptionally low optical energy consumption of ∼25.5 fJ/spike. Importantly, it maintains reliable operation under 95% relative humidity, ensured by a zinc–tin–oxide encapsulation layer, confirming its environmental robustness. Unlike conventional approaches, the WS_2_ memristor simultaneously functions as both sensor and hardware reservoir, exploiting volatile and nonlinear dynamics for direct temporal input processing. Its reservoir encoding capability has been validated on event‐driven datasets with minimal training energy requirements. Beyond task performance, the device demonstrates reproducible electrical and optical characteristics, AC endurance exceeding 1.5 million cycles, and advanced optoelectronic synaptic features including excitatory photo‐synaptic current, short‐term plasticity, long‐term plasticity, and photonic paired‐pulse facilitation. Overall, these findings establish a multifunctional WS_2_ in‐sensor RC platform with superior optoelectronic and multitasking capabilities compared to previously reported WS_2_‐based devices, opening new opportunities for energy‐efficient neuromorphic processing in next‐generation edge technologies.

## 10 Experimental Section

### 10.1 Device Fabrication

The proposed device was fabricated on a silicon (Si) wafer, which was initially cleaned using isopropyl alcohol (IPA) and deionized (DI) water, followed by drying with nitrogen (N_2_) gas. The fabrication began with the growth of a 250 nm thick layer of silicon dioxide (SiO_2_) on the Si wafer using plasma‐enhanced chemical vapor deposition (PECVD) at 400°C. This was followed by the deposition of a 30 nm thick titanium (Ti) layer, serving as an adhesion layer, and a 120 nm thick platinum (Pt) layer as the bottom electrode; both layers were deposited via sputtering at room temperature. Subsequently, a Tungsten disulfide (WS_2_) layer, approximately 100 nm thick, was deposited by drop casting. Next, a 5 nm thick Zn─Sn─O (ZTO) layer was grown as an encapsulation layer using sputtering. Finally, a 100 nm thick copper (Cu) top electrode was deposited using a shadow mask (100 µm in diameter) by RF sputtering in a pure argon (Ar) atmosphere at a pressure of 10 mTorr, completing the Cu/ZTO/WS_2_/Pt memristive device structure.

### 10.2 Characterization and Measurement

The cross‐sectional structure and layer‐by‐layer material composition were analyzed using a high‐resolution transmission electron microscope ((FIB lamella fabrication: Helios G4 FIB/SEM (ThermoFischer Scientific), STEM imaging and EDS elemental mapping: ThemisZ S/TEM microscope working at 300 kV (ThermoFischer Scientific)). X‐ray diffraction (XRD) patterns were recorded with a Bruker D8 Advance diffractometer equipped with nickel‐filtered CuKα (1.5418 Å) radiation. The Raman spectrum was acquired using a WITec Apyron RAMAN spectrometer. X‐ray photoelectron spectroscopy (XPS) was performed on WS_2_ sample under high vacuum using a Kratos Amicus XPS system, equipped with a monochromatic Al Kα X‐ray source operating at 10 kV. The device's electrical characteristics were measured using an Agilent B1500A semiconductor device parameter analyzer. For photoinduced measurements, a visible blue light‐emitting diode source (456 and 532 nm Shanghai Dream Lasers Technology) and electronic shutter controller (Newport) were employed.

## Conflicts of Interest

The authors declare no conflicts of interest.

## Supporting information




**Supporting File**: advs75318‐sup‐0001‐SuppMat.docx.

## Data Availability

The data supporting this article have been included as part of the Supplementary Information.
