# Supplementary material for: WS2 Optoelectronic Memristive Reservoir Enabling Ultra‐Low‐Power, Multi‐Task, and Environmentally Stable Neuromorphic Computing
Source: Adv Sci (Weinh). 2026 Apr 17;13(47):e75318. doi: 10.1002/advs.75318 (PMC13335437; doi:10.1002/advs.75318)
Supplement: Supplementary file 1 [file ADVS-13-e75318-s001.docx]

**WS_2_ Optoelectronic Memristive Reservoir Enabling Ultra-Low-Power, Multi-Task, and Environmentally Stable Neuromorphic Computing**

Dayanand Kumar^1^, Hanrui Li^1^, Divyanshu Divyanshu^2^, Dhananjay D. Kumbhar^1^, Manoj Kumar Rajbhar^1^, Amit Singh^1^, Abdul Momin Syed^1^, Selma Amara^2^, Gianluca Setti^2^, and Nazek El-Atab^1*^

^1^ Smart, Advanced Memory Devices and Applications (SAMA) Laboratory;

^2^ Integrated Intelligent Systems (I2S) Lab;

Electrical and Computer Engineering, Computer Electrical Mathematical Science and Engineering Division, King Abdullah University of Science and Technology (KAUST) Thuwal 23955-6900, Kingdom of Saudi Arabia

^*^Corresponding author: Nazek El-Atab

Email: [nazek.elatab@kaust.edu.sa](mailto:nazek.elatab@kaust.edu.sa)

**Table-S1: Detailed comparison between previously published TMD materials based opto-electronic devices with the current research work**

| **Materials** | **Operation** | **Cycles** | **Energy Consumption** | **Synapse** | **Applications/Accuracy** | **Ref.** |
| --- | --- | --- | --- | --- | --- | --- |
| GeTe/MoTe_2_ | E | 10^5^ | 400fJ | PPC | Pattern Recognition/81.3% | ^[1]^ |
| (PEA)2PbBr_4_ | E | 100 | 30nJ | STDP, EPSC, PPF | Neuromorphic Computing | ^[2]^ |
| h-BN | E | 100 | 125 fJ | STDP/PPF | Reservoir Computing | ^[3]^ |
| PdSeO_x_/PdSeO_2_ | E | 700 | 0.9pJ | LTP/LTD | Image Recognition/ANN: 93% | ^[4]^ |
| PdSe_2_ | E | 100 | 11.25pJ | STDP/PPF | Multipettern Memorization | ^[5]^ |
| Mxene | E | 1000 | 230nJ | STDP/PPF/PPD | Edge Detection/CNN:91% | ^[6]^ |
| MoO_x_ | E+O | 1 | 1050nJ | P/D | Vision System | ^[7]^ |
| MO_2_C/MoS_2_ | E | 100 | 1pJ | PPF/PTP | High Density Memory | ^[8]^ |
| CsPbBr_3_ | E+O | NA | 1.08μJ | PPF | Neuromorphic Vision System | ^[9]^ |
| h-BN/WSe_2_ | E+O | NA | NA | PSC/LTP/LTD and STDP | Pattern recognition/ONN: 90% | ^[10]^ |
| Ga_2_O_3_/MoS_2_ | E+O | 8x10^3^ | NA | LTP/LTD/PPF/PPD/STM/LTM | artificial visual system | ^[11]^ |
| MXene/TiO_2_ | E+O | 10^3^ | NA | P/D/EPSC/PPF/PPD/STDP | Image Edge Detection/CNN: 96% | ^[12]^ |
| α-In_2_Se_2_ | E+O | NA | NA | LTP/LTD/EPSC/IPSC | Vision System ANN: 92.63% | ^[13]^ |
| hBN | E | 2.5×10^6^ | NA | STDP | Image Recognition/ANN: ~88% | ^[14]^ |
| SnS | O | NA | NA | NA | Languate Learning/Reservoir Computing/91% | ^[15]^ |
| WSe_2_ | E+O | NA | 1.03nJ | NA | Neuromorphic Vision System | ^[16]^ |
| MoS_2_ | E+O | NA | 3.6nJ | PPF | Visual Perception | ^[17]^ |
| **WS_2_/ZTO** | **Electrical**  **+**  **Optical** | **1.5×10^6^** | **LTP/LTD/**  **STP/LTP/**  **STM/LTM/**  **PPF** | **2.5 × 10⁻¹⁴ J** | **Digit Recognition~94%**  **MotionPerception~93%**  **Speech Recognition~89%**  **Reservoir Encoder~95%** | **This Work** |

Electric (E), Optic (O), Electric+Optic (E+O), Not Available (NA)

**Note-1: Fabrication Process of Cu/ZTO/WS_2_/Pt structure**

**Figure S1**. Detailed fabrication process of Cu/ZTO/WS_2_/Pt device

The Cu/ZTO/WS_2_/Pt device was fabricated on a silicon substrate. For device fabrication, the Si sample was initially cleaned using a standard cleaning process. Then, a 250 nm SiO_2_ film was deposited by plasma-enhanced chemical vapor deposition (PECVD) at 400°C. Following this, a 30 nm Ti adhesion layer and a 120 nm Pt layer as a bottom electrode (BE) were deposited by sputtering at room temperature. Subsequently, a WS_2_ thin film was applied using the drop cast method and dried at 60°C until dry (WS_2_ solution was purchased from 2D semiconductors). Afterward, a 5 nm Zn_2_SnO_4_ (ZTO) diffusion barrier layer was deposited by sputtering at room temperature. Finally, a 100 nm Cu layer was deposited by sputtering using a shadow mask to form the complete Cu/ZTO/WS_2_/Pt structure.

**Note-2: WS_2_ Thin Film Quality and Thickness Control**

The WS_2_ layer thickness (~100 nm) was controlled by regulating both the concentration of the WS_2_ dispersion and the volume deposited during the drop-casting process. The WS_2_ solution used without changing existing concentrations (standard:1mg/mL). Multiple trials were performed to calibrate the concentration–volume correlation, ensuring reproducibility across all fabricated devices. The WS_2_ solution was purchased from 2D Semiconductors company.

**Figure S2a** shows the SEM micrographs of drop-casted WS_2_ thin film on Si. A large area coverage of the flakes with a percolative network can be clearly observed from the SEM. The SEM analysis provides critical insight into the film's lateral continuity and overall morphology. Specifically, the images confirm that the WS_2_ nanosheets interlock to form continuous pathways across the substrate surface. The SEM confirms the microscopic coverage and long-range connectivity of the WS_2_ flakes. Achieving uniform WS_2_ thin-film coverage across a full 4-inch wafer is currently not feasible with the drop-casting technique. Figure S2b, c presents a three-dimensional AFM height map acquired over a 10 × 10 µm² area of the drop-cast WS_2_ thin film on Si, with horizontal line-scan profiles extracted from random locations across the scanned region. The AFM topography shows a continuous but morphologically heterogeneous film composed of nanoscale WS_2_ islands and interconnected clusters distributed across the Si substrate. The measured height variation spans approximately −0.02 to 0.17 µm, with a root-mean-square (RMS) roughness of 16.72 nm, indicative of a rough but percolative thin-film morphology. The line-scan profiles also agree with the AFM height map, showing repeated thickness fluctuations across the full lateral scan length (Figure S2c). Importantly, no extended bare Si regions are observed in either the height map or the line profiles, confirming that WS_2_ covers the substrate across the scanned area despite the non-planar morphology. Such thickness variations are characteristic of solution-processed, drop-casted films, where nanosheet stacking, partial restacking, and solvent evaporation dynamics lead to spatially varying film thickness rather than atomically flat layers. From a device perspective, the nanoscale height variations and inter flake junctions introduce a high density of grain boundaries, sulfur vacancies, edge sites, and interlayer interfaces. These features act as charge trapping and ion migration sites, which are well known to facilitate memristive and synaptic behaviors in TMD-based neuromorphic devices. While the WS₂ film is not crystalline or uniform in the epitaxial sense, it however provides a defect-rich, electrically percolated network that is highly suitable for synaptic modulation.

**Figure S2.** (a) Scanning electron microscopy (SEM) micrograph of WS_2_ layer. (b) 3D mapping of a 10 um^2^ area using atomic force microscopy (AFM). (c) The line scan thickness profile over random areas across the scan area.

**Note-3: I-V Characteristics of WS_2_ and ZTO Devices.**

Figure S3. (a) I-V characteristics of Cu/WS_2_/Pt device for continuous over 1000 cycles. (b) DC cycling endurance of the Cu/WS_2_/Pt device. (c) I-V characteristics of the Cu/ZTO/Pt device.

We initially fabricated two single-layer devices: Cu/WS_2_/Pt and Cu/ZTO/Pt, as shown in Figure S3. Figure S3a presents the current–voltage (I–V) characteristics of the Cu/WS_2_/Pt device measured over 1000 continuous cycles. Figure S3b shows the DC endurance of the device for 1000 cycles with was measured at read voltage of 0.1 V. The device exhibited highly unstable switching behavior in both low-resistance state (LRS) and high-resistance state (HRS), along with significant fluctuation in the switching voltages. This instability makes the single-layer WS_2_ configuration unsuitable for reliable memory or neuromorphic computing applications. Similarly, Figure S3c shows the I–V curve of the Cu/ZTO/Pt device, which failed to exhibit any distinguishable memory window. The absence of bistable behavior is primarily attributed to the extremely thin ZTO layer (~5 nm), which limits charge trapping and storage capacity. Consequently, neither the WS_2_-only nor the ZTO-only device demonstrated stable resistive switching behavior required for practical applications.

To overcome these limitations, we engineered a heterostructure bilayer configuration (ZTO/WS_2_) that combines the complementary advantages of both oxides and transition metal dichalcogenides. The resulting Cu/ZTO/WS_2_/Pt device exhibited stable, repeatable, and reproducible characteristics under both electrical and optical stimuli. These results, thoroughly discussed and analyzed in the main manuscript, confirm that the ZTO/WS_2_ bilayer architecture provides the optimal structural and electronic configuration for multifunctional and neuromorphic applications.

**Note-4: Electrical performance of the Cu/ZTO/WS_2_/Pt devices**

**Variability and reliability of the devices**

**Figure S4**. a) Cumulative distribution function (CDF) of continuous switching cycles. b-c) Distribution of the ON and OFF state currents. d) High temperature stability of the device.

The electrical characteristics of Cu/ZTO/WS_2_/Pt device were demonstrated in **Figure S4**. **Figure S4a** presents the cycle-to-cycle cumulative distribution function (CDF) of 1.5 M switching cycles, demonstrating consistent variability of the device. This observation further reinforces the stability and reliability of the device across varying operational cycles. We conducted a statistical analysis of the ON and OFF state currents of the device, as shown in **Figure S4b-c**, where histograms illustrate distributions fitted to a normal distribution. The analysis reveals a concentrated distribution for the ON state at approximately 0.00728 A and for the OFF state at around -8.019E-5 A, indicating a stable operational range. The standard deviations (σ) for the ON and OFF states are 0.00141 A and 4.724E-5 A, respectively. This consistency in ON/OFF state currents underscores the device's reliable and consistent performance. To check the high temperature stability of the device, we measured our device at 100°C, as shown in **Figure S4d**. The results confirm that the device’s LRS and HRS state are stable and has capability at high temperature operation. These results confirm that the Cu/ZTO/WS_2_/Pt devices are capable for industrial applications in near future.

**Figure S5** a) Stability of 100 consecutive sweeping I-V curves. b) and c) Endurance performance over 100 cycles, with a focus on the extracted parameters V_SET_, I_SET_, V_RESET_, and I_RESET_ from the I-V characteristics.

The stability assessment was performed by analyzing 100 consecutive sweeping I-V curves, depicted in **Figure S5a**. These results emphasize the robust cycle stability and repeatability of the device, as evidenced by the consistent behavior of the I-V curves across increasing sweep cycles. Additionally, **Figure S5b-c** illustrate the endurance performance over 100 cycles, focusing on the extracted parameters V_SET_, I_SET_, V_RESET_, and I_RESET_ from the I-V characteristics. The proposed memristive device demonstrates reproducibility, maintaining variability within acceptable ranges as compared to previous studies

**Figure S6** The histograms display the distributions fitted using the Normal distribution statistical count accurately represents **a)** V_SET_ and **b)** V_RESET_, **c)** I_SET_ and **d)** I_RESET_

The cyclic reliability statistically assessed for the SET and RESET voltages, along with their corresponding currents (**Figures S6**), where histograms illustrate distributions fitted to a normal distribution. These analyses accurately represent V_SET_ and V_RESET_ within acceptable ranges. The data shows a concentrated distribution of V_SET_ at approximately 0.71 V and V_RESET_ at around -0.58 V, with I_SET_ and I_RESET_ measured at roughly 1.7E-4 A and -0.082 A, respectively, indicating a stable operational range. The standard deviations (σ) for V_SET_ and V_RESET_ are recorded at 0.14 V and 0.066 V, respectively, while those for I_SET_ and I_RESET_ are 8.59E-4 A and 0.0034 A, respectively. This consistency in operational voltages and currents provides a strong foundation for the practical application of the device.

**Figure S7** Cyclic variability in 12 devices I-V sweeps across randomly chosen 24 devices [D1-D12]

**Figure S8** Cyclic variability in 12 devices I-V sweeps across randomly chosen 24 devices [D13-D24].

To thoroughly evaluate the reliability and reproducibility of devices across multiple fabricated arrays on the wafer, a set of randomly selected devices underwent extensive testing through consicutive 100 cycles. The outcomes from 24 I-V cycles of these devices, as shown in **Figures S7** and **S8**, provide valuable insights into their performance. These results reveal the cycle-to-cycle (C2C) and device-to-device (D2D) variations in SET and RESET voltages during the continuous switching cycles, but this variability is acceptable in filamentary based memristors. These results are suitable for ensuring predictable device behavior, establishing a solid foundation for their practical application in various scenarios

**Figure S9**.Cumulative distribution functions (CDFs) for both set and reset operations. (a) CDF with V_SET_ and V_RESET_. (b) CDF with I_SET_ and I_RESET_. (c) CDF with I_ON_ and I_OFF_.

As shown in **Figure S9a** and **S9b**, the statistical analysis of cycle-to-cycle variability is presented in terms of cumulative distribution functions (CDFs) for both SET and RESET operations with voltages and currents. The CDF plots reveal a clear and well-defined separation between the switching parameters extracted from the SET and RESET events, indicating stable and repeatable resistive switching behavior. The distinct distribution profiles of current and voltage for the two switching polarities confirm the absence of significant overlap, which is essential for reliable device operation in memory and neuromorphic applications.

In particular, the ON-state (I_ON_) and OFF-state (I_OFF_) currents exhibit a pronounced separation window (**Fig.S9c**) The I_OFF_ state shows a mean and median value of approximately 1.0 × 10^-4^ A, demonstrating a tightly confined distribution with minimal dispersion. In contrast, the I_ON_ state exhibits a mean current of 7.3 × 10^-3^ A and a median of 7.4 × 10^-3^ A, with a standard deviation of 1.5 × 10^-3^ A. The close agreement between mean and median values for both states indicates a nearly symmetric distribution without significant skewness, suggesting stable filament formation and rupture dynamics across switching cycles. Importantly, the resulting ON/OFF current ratio is on the order of ~10^2^, providing a sufficiently large sensing margin for read operations and reducing the probability of read errors due to state overlap. The relatively moderate standard deviation in the ON state further suggests controlled conductive filament growth with limited stochastic fluctuations, while the narrow OFF-state distribution reflects effective filament rupture and high-resistance stability. Overall, the statistical separation observed in the CDF analysis confirms switching uniformity, low cycle-to-cycle variability, and a well-defined resistive window, which are critical parameters for scalable hardware implementation and large-scale array integration.

**Note-5: Voltage pulse schemes for emulating synaptic functions.**

**Figure S10.** Pulse schemes applied to the synaptic memristor for achieving potentiation and depression behaviors.

Here, Gmin, and Gmax are minimum and maximum conductance of the device, respectively. Pmax represents the maximum pulse number. These parameters are extracted from the experimental data and impetrated to switch the device. and B is just a function of A that fits the functions within the ranges of Gmax, Gmin, and Pmax.

**Note-6: Branching Point Between STP to LTP**

As illustrated in Figure 5b (main manuscript), the transition from STP to LTP is examined under varying numbers of stimulus pulses, ranging from 5 to 25. As observed in **Figure S11a–e**, following the application of 5 stimulus pulses, the PSC returned to its initial state within 25 minutes. In contrast, for 10 pulses, a significant amount of current was retained for up to 30 seconds. Similarly, for 15, 20, and 25 pulses, a pronounced retention of response was observed, persisting for at least 30 seconds. Notably, the transition from STP to LTP in this case necessitates a systematic investigation into the decay dynamics of the PSC. To elucidate this decay behavior, the PSC decay following stimulus removal was plotted and fitted using the exponential dec 2 model in Origin, as depicted in **Figure S11f**. Additionally, two critical characteristics—weight gained (or retained) and the decay time constant (τ_2_) are presented in **Figure S12**. The weight gained from stimulus pulses (ranging from 5 to 25) is clearly evident in **Figure S12a**, demonstrating an increase from 15% to 21.58%, calculated using the formula ((𝐼_𝑛_−𝐼_𝑜_)/𝐼_𝑜_)×100)). Meanwhile, the retained weight for 5 to 25 pulses ranged from 0% to approximately 4.5%. It is important to highlight that both weight gain and weight retention exhibited a linear dependence on the number of stimulus pulses. Subsequently, an exponential non-linear fit was applied to the decayed PSC, and the extracted τ_2_ values are presented in **Figure S12b**. For 5, 10, and 15 pulses, the retained PSC exhibited relatively small and comparable τ_2_ values. However, for 20 and 25 pulses, τ_2_ values were noticeably larger, increasing in a non-linear manner. These observations indicate that, in terms of STP-to-LTP transition, 20 or more pulses may serve as the critical threshold at which the PSC response shifts from STP to LTP.

**Figure S11.** (a–e) illustrate the excitatory post-synaptic current (EPSC) behavior of the device under 5, 10, 15, 20, and 25 stimulus pulses, as well as the retention of the PSC after stimulus removal. (f) presents the decay characteristics of the PSC for each stimulus condition, fitted using a standard exponential model.

**Figure S12**. (a) Weight retained by the device for each pulsing scheme after 30 seconds. (b) τ_2_ values obtained from the exponential dec 2 model after fitting the decay for each stimulus condition.

**Note-7: Optical Characteristics of Cu/ZTO/WS_2_/Pt device prior to the electroforming.**

**Figure S13**: (a) EPSC response of the device under 30 consecutive spikes with a pulse width of 0.5 s and an interval time of 0.5 s, using a light intensity of 4.2 mW/cm². (b) EPSC response of the device under 1 s light stimulation (4.2 mW/cm²) at wavelengths of 456 nm and 532 nm. (c) EPSC response of the device under 30 consecutive spikes with a pulse width of 0.5 s and an interval time of 0.5 s, using a light intensity of 92 mW/cm². (d) EPSC response of the device under 1 s light stimulation (4.2 mW/cm²) at wavelengths of 456 nm and 532 nm.

We examine optically stimulated synapse under two conditions, first one is prior to the electrical forming and second one is after electrical forming and multiple switching cycles. As depicted in **Figure S13**, **Figure S13(a-b)** represents the optical synapse of the device prior to the electrical forming while **Figure 13(c-d)** represents the optical synapse after electrical forming. Under both conditions devices were capable to produce synaptic responses in the form of EPSC, whereas it needs to note that after forming devices, it requires higher optical stimuli power/intensity to overcome HRS level and show synaptic responses while in the observation of prior to the electroforming, showing better and reliable synaptic functionality at lower power for the same wavelength and pulsing parameters. As the main aim of this work to produce low energy synaptic functionalities we explored the devices prior to the electroforming.

**Figure S14** depicts the optical characteristics of the device for short-term plasticity (STP) to long-term plasticity (LTP). a) conversion of STP to LTP of the device for the various wavelength intensities from 1.2 mW/cm2 to 4.2 mW/cm^2^ for 456 nm wavevength. b) STP to LTP conversion at various time with fixed light intensity of 4.2 mW/cm^2^. c) Learning forgetting and relearning behavior process of the device.

We further examined the transformation from STP to LTP by adjusting the illumination time as well as intensity of light, as shown in **Figure S14a** and **S14b**. A longer illumination time correlates with a higher EPSC and a slower decay rate. In essence, the EPSC response of the device can be transitioned from STP to LTP by either manipulating the light intensity or the duration of illumination. This indicates a powerful capability in replicating superior memory functions for visible light.^[7, 18]^ We have also depicted the learning and forgetting process in our device by cyclically turning the light ON and OFF, as shown in **Figure S14c**. In this case, turning ON the light represents the learning and relearning behavior in the device, while turning OFF the light symbolizes the forgetting behavior. The EPSC of the device increases with light exposure and then diminishes to an intermediary level after a specified period, suggesting that the learned information gradually fades over time. However, after repeatedly cycling through the learning or relearning process, the photocurrent conductance of the device gradually increases (A5 > A4 > A3 > A2 > A1), implying that previously learned information can significantly enhance memory capability. The photocurrent conductance of the device reaches its peak level (A5) after 5 cycles of learning and relearning, indicating a transition from short-term memory (STM) to long-term memory (LTM). This excellent repeatability in photocurrent response facilitates the simulation of superior synaptic function in synaptic plasticity, as illustrated by the "learning-forgetting-relearning" process.^[19]^

**Optically Operatable Device to Device Uniformity**:

**Figure S15.** (a) Device to device (D2D) variability across 12 devices (b) I_retained_ values and (c) Variation in the dark currents across nine devices.

Prior studies employing PVD-deposited thin films^[20]^ where thickness control is significantly more precise than drop casting, have demonstrated that even small variations in film thickness which led to measurable, though minimal, device to device (D2D) and cycle-to-cycle (C2C) variability in synaptic behavior (**Figure S15a**). The transient responses, I_Retained_ and dark current across Devices 1–5, as well as repeated cycling in Device 6, remain largely consistent. This observation indicates that minimal degree of variability is a characteristic to opto-synaptic devices developed using highly uniform PVD-grown thin films. In the present study, direct thickness measurement of the WS_2_ layer for every individual device using high-resolution transmission electron microscopy (HRTEM) was not practically feasible, as such analysis is destructive, time-consuming, and cannot be routinely performed across large device populations. Instead, to reliably assess thickness uniformity and its impact on device performance, we carried out a comprehensive **D2D variation analysis** using **nine randomly selected devices fabricated at different locations across the same sample (Figure S15b).** This statistical approach is widely adopted in memristive and neuromorphic device studies as a realistic indicator of film uniformity and process reproducibility. The measured retained current EPSC (I_retained_) and dark current values exhibit, indicating a high degree of uniformity in the drop-cast WS_2_ film and consistent device fabrication conditions (**Figure S15c)**. Importantly, the observed variability remains well within the acceptable range commonly reported for filamentary and defect-assisted memristive devices, where slight local differences in film thickness, grain boundaries, and defect density are unavoidable. Although the **absolute photocurrent magnitude shows modest device-to-device fluctuations**, which can be attributed to local thickness-dependent optical absorption and variations in light–matter interaction volume.

**Note-8: Optical Characteristics of Cu/ZTO/WS_2_/Pt device for 532 and 635 wavelength**

**Figure-S16**. a) EPSC of the device for 532 nm wavelength with 4.2 mW/cm^2^ intensity and different pulse width and different interval time. b) EPSC of the device at various illumination time with fixed intensity of 4.2 mW/cm^2^. c) EPSC of the device at various light intensity with fixed illumination time. d) EPSC of the device for 635 nm wavelength with 4.2 mW/cm^2^ intensity and different pulse width and different interval time. e) EPSC of the device at various illumination time (1s, 2s, 3s, 4s, and 5s) with fixed intensity of 4.2 mW/cm2. **f** EPSC of the device at various light intensities (1.7, 2.5, 3.4 and 4.2 mW/cm^2^) with fixed illumination time of 1s.

Similar to 456 nm wavelength, device also demonstrated the optical synaptic characteristics of the Cu/ZTO/WS_2_/Pt device for 532 nm wavelength, which are depicted in **Figure S16**. **Figure S16a** shows the excitatory postsynaptic current (EPSC) triggered by an optical spike as a function of the spike duration (0.1s and 0.5s) under the same light intensity of 4.2 mW/cm^2^. The results confirm that the EPSC increase with increasing the pulse duration. The transition from STP to LTP by varying both the duration and intensity of light exposure, as illustrated in **Figure S16b** and **S16c**. It was observed that a longer duration of illumination is associated with an increased excitatory postsynaptic current (EPSC) and a reduced decay rate. Essentially, the EPSC response of the device can be shifted from STP to LTP by adjusting either the intensity or the duration of the light exposure when 532 nm wavelength was illuminated. This demonstrates the device's ability to mimic advanced memory functions under visible light.

**Note-9: Mechanisms Validation for Optoelectronic Devices**

**Figure S17**. a)The top and side views of the optimized pristine WS2 structure are shown, where tungsten (W) atoms are represented by brown spheres and sulfur (S) atoms by yellow spheres. The blue outline highlights the 2 × 2 supercell employed in the calculations. Electronic band structure of monolayer, b) WS_2_, c) WS_1.75_, d) WS_1.75_Cu_0.25_ plotted along high-symmetry directions Γ → M → K → Γ. The Fermi level set at 0 eV. Blue dots represent calculated energy levels, and dashed lines indicate high-symmetry transitions.

**Calculation details:**

All the calculations were performed using Quantum ESPRESSO package.^[21]^ We first ran the structural relaxation for a system composed of tungsten (W) and sulfur (S), containing a total of 12 atoms, to obtain the most stable energy configuration. The calculation was set up to optimize both atomic positions and cell parameters using variable-cell relaxation. The custom lattice was explicitly defined using three vectors, with a notably large c-axis of 29.2 ˚A, indicating a monolayer geometry. Electronic structure settings included a wavefunction cutoff energy of 35 Ry and a charge density cutoff of 280 Ry. Spin polarization was enabled for both atomic species, with initial magnetizations set to 0.10 for sulfur and approximately 0.36 for tungsten. Occupations were treated using cold smearing with a degauss value of 0.0125 Ry, which is suitable for metallic or magnetic systems. The relaxation was guided by a force convergence threshold of 1.0 × 10^−4^ Ry/Bohr and an electronic convergence threshold of 2.4×10^−9^ Ry. A maximum of 80 self-consistent field (SCF) steps was allowed, with a mixing beta of 0.4 to stabilize convergence. The Brillouin zone was sampled using a dense 12 × 12 × 1 Monkhorst-Pack grid, appropriate for capturing in-plane interactions in layered materials while assuming minimal dispersion along the c-axis. The exchange- correlation energy of electrons is treated with a generalized gradient approximation (GGA) as formulated in the energy functional of Perdew, Burke, and Ernzerhof (PBE).^[22]^

**Mechanism for optoelectronic device:**

The pristine WS₂ (**Figure S17a**) possesses a direct band gap of approximately 1.8 eV at the K-point of the Brillouin zone (**Figure S17b**). This direct band gap enables efficient photon absorption and electron–hole pair generation under light illumination, resulting in enhanced carrier density and increased channel conductance. Consequently, the optoelectronic behavior of the device is primarily governed by the photoconductive effect of the ZTO/WS_2_ heterostructure, where photoexcited carriers contribute to synaptic-like conductance modulation. When sulfur vacancies are introduced into WS₂, the electronic structure undergoes significant modification (**Figure S17c**). The band gap decreases to about 0.78 eV and becomes indirect, while mid-gap defect states emerge within the band gap region (**Figure S17c**). These localized states act as charge trapping centres, which prolong carrier lifetimes and lead to persistent photoconductivity even after the light source is removed. This defect-assisted mechanism explains the gradual decay of photocurrent and the light-induced conductance increase observed experimentally. Importantly, sulphur vacancies do not form conductive filaments; rather, they alter the electronic band structure and carrier dynamics, enabling optical modulation without filamentary conduction.

**Mechanism for ECM device:**

The incorporation of Cu atoms into the WS₂ lattice modifies its electronic structure, as confirmed by the calculated band diagram for WS_1.75_Cu_0.25_. Unlike pristine WS₂, which exhibits a semiconducting band gap, the Cu‑doped system displays multiple states crossing the Fermi level, indicating metallic behavior (**Figure S17d**). This metallicity arises from strong hybridization between Cu orbitals and the WS₂ lattice, which introduces delocalized states near the Fermi energy and eliminates the band gap. These delocalized states act as continuous conduction pathways that support the electrochemical metallization process described in Figure S7.

Under positive bias, Cu atoms at the top electrode undergo oxidation, releasing Cu⁺ ions that migrate through the ZTO/WS₂ switching layer. As these ions approach the bottom electrode, they are reduced to form metallic Cu clusters. The presence of Cu‑induced metallic states lowers the energy barrier for electron transfer, thereby facilitating filament nucleation and growth. As the conductive filament extends and bridges the electrodes, the device transitions into a low‑resistance state (LRS). Once formed, the metallic nature of the Cu‑doped WS₂ ensures efficient electron transport through the hybridized states.

When the bias is reversed, Joule heating and electrochemical reactions rupture the filament at its narrowest point, restoring the high‑resistance state (HRS). This switching mechanism is consistent with the band structure analysis: Cu‑induced metallic states explain both the ability of the device to sustain filamentary conduction and the stability of its resistive switching. In summary, Cu incorporation transforms WS₂ from a semiconductor into a metallic conductor, providing the theoretical basis for electrochemical filament formation and enabling reliable resistive switching suitable for neuromorphic applications

**Note-10: Humidity testing setup and PPF ratio with time at 95% humid condition**

**Figure S18**. a) Humidity testing setup (Nextron Korea) during device measurements. b) PPF ratio of the device was tested at various time interval form 5 to 50 hours with 95% of humidity level. We found minor or negligible difference in PPF values confirming that the device can perform well in longer humid condition.

The measurements were performed in a programmable relative-humidity chamber (Nextron, Korea) equipped with closed-loop RH and temperature control. The RH was set between 20 and 95% with an accuracy of approximately ±1% RH, and the chamber temperature was maintained at room temperature (22.5 °C) to eliminate temperature-induced drift in synaptic responses. For each RH setpoint, the device was first kept under the target humidity for a stabilization dwell time of 5 min to ensure equilibrium adsorption. All electrical and optoelectronic synaptic measurements were then conducted in situ using electrical feedthroughs without removing the sample from the chamber, and the measurement duration at each RH level was kept constant to enable fair comparison across conditions.

**Note-11: Multi-tasking Features Description**

**Figure S19. (a)** EPSC of the device with the pulse width of 0.1s and interval time of 0.1s for 5, 10, 15, 20, 25 spikes, respectively. **(b)** Corresponding fitting parameter for 25 Spikes data used for WS_2_ reservoir Kernel.

**Motion-Perception using Synthetic Ball Movement Dataset**

To validate the effectiveness of the WS_2_ inspired reservoir for motion perception, we created a synthetic Ball Motion dataset that simulates five classes of controlled directional movement in a 2D frame space: Static, Left, Right,Up, and Down. Each sample consists of a 12-frame video (32*32 pixels) where a circular object with radius of 5 pixels moves in a consistent direction. The movement vector is defined by a class specific delta (dx, dy) and randomized speed (1-2 pixels/frame). The starting co-ordinates are dynamically selected to ensure the object remains within bounds during motion, preventing edge clipping. Each class contains 150 samples. After generating the raw frames, ON/OFF spikes are computed as the difference between consecutive frames, mimicking the working principle of event-based vision sensors. These spatiotemporal spikes are then passed through a WS_2_ inspired reservoir, enabling robust response generation to distinguish fine-grained directional dynamics. **Figure S20** shows sample frames from the generated synthetic ball motion dataset, while **Figure S21a** presents the 2D t-SNE projection of the extracted reservoir features. For each pixel, five features are computed from the reservoir trace: total energy, peak value, final value, time of peak, and weighted spike timing. These are flattened across the 32×32 spatial grid, resulting in a total of 5120 features (32 × 32 × 5) per sample. These features become the readout layer input to an MLP Classifier. To further compare the behavior of the reservoir-enabled approach, a baseline ANN is used, which only uses the last frame of each input video (flattened pixels) to classify motion, without any temporal or reservoir processing. With a uniform training strategy, it obtains an accuracy of about ~58% as compared to ~93% of Reservoir MLP. **Figure S21b** shows the comparison of both the classifier.

 **Figure S20.** 12 frames for each class is algorithmically generated for motion trajectories.

**Figure S21. (a)** t-SNE projection of reservoir feature. **(b)** Reservoir MLP vs Baseline ANN comparison.

**FSDD- Free Spoken Digit Dataset- Speech Recognition Task**

FSDD is an open-source audio dataset containing short recordings of spoken digits from 0-9, by multiple speakers in English. They are typically recorded at 8 kHz. The raw ‘.wav’ files from the dataset are converted into spike-like 1D temporal signals, and the frequency domain MFCC (Mel Frequency Cepstral Coefficients) features are extracted. **Figure S22** shows the MFCC features of all the digit classes. These inputs are then passed through the WS_2_ reservoir Kernel to simulate fading memory dynamics. From the resulting reservoir response, a total of 21 features are extracted (temporal descriptors and statistical summaries), to capture the temporal dynamics and shape characteristics of the trace, as shown in **Figure S23**. These are concatenated with MFCCs to form a hybrid spatio-temporal feature vector for each sample (13 MFCC features and 21 Reservoir features). These features become the readout layer input to an MLP Classifier and follows the unified approach mentioned in the main text.

**Figure S22.** MFCC Feature for all the digit class to represent audio frequency characteristics.

**Figure S23.** Heatmap for Mean feature values for each digit class vs different extracted features.

**N-MNIST- Event based spike recording**

Following the same unified framework, the N-MNIST dataset is first loaded in binary format and reconstructed into 3D spike matrices (Time* Height* Width). Each spike train per pixel is passed through the Reservoir kernel. From the resulting spatiotemporal reservoir trace, five temporal features per pixel are extracted: total energy, maximum response, final value, time of peak, and weighted spike timing. These features are flattened into a high-dimensional vector (5 × 34 × 34 = 5780 features) per sample and used as input to the MLP. **Figure S24a** shows the Reservoir feature across different digit class, and as each digit class has a mean feature vector derived from the reservoir trace, cosine similarity matrix gives a measure of how similar the direction of these mean vectrors are as shown in **Figure S24b**.

**Figure S24.** (a) Heatmap for Mean Reservoir feature values for each digit class. (b) Cosine Similarity Matrix.

The double-exponential kernel parameters were extracted by fitting the experimentally measured EPSC decay curves (single device) shown in **Fig. 5b** and Supplementary Fig. S16b using a nonlinear regression approach. The extracted parameters were directly implemented in the reservoir model without further modification. These values are explicitly implemented in all reservoir simulations (i.e., the kernel is fixed for all tasks). No task-specific tuning or optimization was performed. This ensures that the demonstrated performance originates from intrinsic device temporal dynamics rather than algorithmic parameter adjustment.

For all datasets, the training pipeline was unified as follows:

•	Train/test split: 80/20 (stratified)

•	Random seed: 42 (fixed) to ensure reproducibility of the reported results.

•	Classifier: MLP readout only

•	Hidden layers: (128, 64)

•	Training epochs: 30

•	Reservoir weights: fixed (non-trainable)

To assess statistical robustness, the simulation was repeated across five independent random seeds (1, 10, 21, 42, 77). This information is added in Supplementary Table S2. The low standard deviation (<1.5% for all tasks) confirms stable convergence and robustness of the implemented framework. The reported accuracy corresponds to the final epoch performance.

To assess statistical robustness, the simulation were repeated across five independent random seeds (1, 10, 21, 42, 77).

**Table S2: Accuracy variation across independent random seeds**

| Task | Obtained Accuracy (%) for Seed Value | | | | | Mean Accuracy (%) | S.D. (%) |
| --- | --- | --- | --- | --- | --- | --- | --- |
|  | 1 | 10 | 21 | 42 | 77 |  |  |
| Motion | 92 | 90.67 | 90 | 93.33 | 93.33 | 91.87 | 1.36 |
| Speech | 88.67 | 89.33 | 88.33 | 88.33 | 87.00 | 88.33 | 0.76 |
| Digit | 94.90 | 95.25 | 93.90 | 94.10 | 94.55 | 94.44 | 0.46 |

**Note-12: Simulation description**

The experiment is based on neuromorphic dataset NMNIST^[23]^, which is a variant of traditional MNIST dataset captured by DVS camera. It transforms the static images into spike patterns that mimic the way biological neurons process information. Spike polarity can be interpreted as the optical stimulus to pair of devices while non-spike state represents constant time decay with inherent device property. Therefore, the event-based data can be encoded and extracted by our energy-efficient reservoir layer. The single-layer ANN-based encoder adopts a 1156×10 architecture, while the two-layer ANN features a 1156-128-10 structure, both operating across multiple time steps. Each input frame, a 34×34 pixel event-based snapshot, is processed sequentially to capture temporal dynamics efficiently.

**Figure S25.** a) Visualization of asynchronous of raw spike events with label “3”. b) Visualization of synchronous frame-like event representation.

The event-to-frame integrating method for pre-processing neuromorphic datasets is used in the DVS dataset preprocessing.^[24]^ The method helps split number of events in each slice and integrate the sparse event-based data. The data visualization is shown in **Figure S25a**. Figs represents the asynchronous raw spike event and **Figure S25b** presents the data after prepossessing.

**Event Processing hardware implementation**

The NMNIST dataset utilizes a Dynamic Vision Sensor (DVS) camera, which encodes changes in light intensity as ON and OFF events, representing positive and negative polarity spikes, respectively. In our proposed system, the WS₂ memristor exhibits optical synaptic behavior with positive polarity only; however, additional circuitry using paired devices can be implemented to interpret both event types. The **Figure S26** shows the differential encoding method for proposed memristor device, where pairs of memristors represent complementary excitatory and inhibitory synaptic responses. Two transimpedance amplifiers (TIAs) convert current to voltage, while a differential comparator subtracts the voltages from paired devices for event processing. The extracted feature is read by sample-and-hold (S/H) block and fed into memristor crossbar for further classification.

**Figure S26.** Proposed hardware implementation for dual event preprocessing and memristor read out circuit.

For the event-driven N-MNIST demonstration, the dataset is generated using a Dynamic Vision Sensor (DVS) camera, which encodes temporal changes in light intensity into asynchronous ON and OFF events. In our proposed system, the WS_2_ memristor exhibits optical synaptic behavior with conductance change. Namely, optical stimulation induces conductance potentiation, mimicking excitatory synaptic behavior. Since the device does not intrinsically support symmetric bidirectional (positive/negative) optical modulation, additional peripheral circuitry is introduced to enable full polarity interpretation for N-MNIST processing.

As illustrated in **Figure S26**, a differential encoding scheme using paired memristor devices is adopted. In this configuration:

- One memristor in the pair represents the excitatory (ON event) pathway.
- The complementary memristor represents the inhibitory (OFF event) pathway.
- Both ON and OFF events are driven with the same optical stimulus.

Although each individual WS_2_ memristor responds with same optical stimulus, event polarity is distinguished at the system level by routing ON and OFF events to different devices within the pair. This effectively emulates complementary excitatory and inhibitory synaptic responses.

**For signal readout and hardware mapping:**

1. Each memristor produces a photocurrent proportional to its conductance state.
2. Two transimpedance amplifiers (TIAs) convert the photocurrents from the paired devices into corresponding voltages.
3. A differential comparator subtracts the two voltages, generating a signed output that reflects the net synaptic response: $V_{out} \propto(V_{on}- V_{off})$

This differential operation reconstructs polarity information at the circuit level, even though each device individually exhibits only positive optical plasticity. The resulting differential signal represents the extracted feature corresponding to the DVS event stream. This signal is then captured by a sample-and-hold (S/H) block to stabilize the temporal output and forwarded to the memristor crossbar array for weighted summation and final classification.

**Reference**

[1]	A. C. Khot, K. A. Nirmal, T. D. Dongale, T. G. Kim, *Small* **2024**, 20, 2400791.

[2]	H. Tian, L. Zhao, X. Wang, Y.-W. Yeh, N. Yao, B. P. Rand, T.-L. Ren, *Acs Nano* **2017**, 11, 12247.

[3]	G. H. Zhang, J. R. Qin, Y. Zhang, G. D. Gong, Z. Y. Xiong, X. Y. Ma, Z. Y. Lv, Y. Zhou, S. T. Han, *Adv Funct Mater* **2023**, 33.

[4]	Y. Li, S. Chen, Z. Yu, S. Li, Y. Xiong, M. E. Pam, Y. W. Zhang, K. W. Ang, *Advanced Materials* **2022**, 34, 2201488.

[5]	Y. S. Li, L. Loh, S. F. Li, L. Chen, B. C. Li, M. Bosman, K. W. Ang, *Nat Electron* **2021**, 4, 348.

[6]	N. B. Mullani, D. D. Kumbhar, D. H. Lee, M. J. Kwon, S. y. Cho, N. Oh, E. T. Kim, T. D. Dongale, S. Y. Nam, J. H. Park, *Advanced Functional Materials* **2023**, 33, 2300343.

[7]	F. C. Zhou, Z. Zhou, J. W. Chen, T. H. Choy, J. L. Wang, N. Zhang, Z. Y. Lin, S. M. Yu, J. F. Kang, H. S. P. Wong, Y. Chai, *Nat Nanotechnol* **2019**, 14, 776.

[8]	X. Tang, L. L. Yang, J. H. Huang, W. J. Chen, B. H. Li, S. D. Yang, R. L. Yang, Z. P. Zeng, Z. K. Tang, X. C. Gui, *Npj Flex Electron* **2022**, 6.

[9]	Q. B. Zhu, B. Li, D. D. Yang, C. Liu, S. Feng, M. L. Chen, Y. Sun, Y. N. Tian, X. Su, X. M. Wang, S. Qiu, Q. W. Li, X. M. Li, H. B. Zeng, H. M. Cheng, D. M. Sun, *Nat Commun* **2021**, 12.

[10]	R. Jana, S. Ghosh, R. Bhunia, A. Chowdhury, *J Mater Chem C* **2024**, 12, 5299.

[11]	R. L. Li, W. X. Wang, Y. Li, S. Gao, W. J. Yue, G. Z. Shen, *Nano Energy* **2023**, 111.

[12]	N. B. Mullani, D. D. Kumbhar, D. H. Lee, M. J. Kwon, S. Cho, N. Oh, E. T. Kim, T. D. Dongale, S. Y. Nam, J. H. Park, *Adv Funct Mater* **2023**, 33.

[13]	G. C. Wu, L. Xiang, W. Q. Wang, C. D. Yao, Z. Y. Yan, C. Zhang, J. X. Wu, Y. Liu, B. Y. Zheng, H. W. Liu, C. W. Hu, X. X. Sun, C. G. Zhu, Y. Z. Wang, X. Xiong, Y. Q. Wu, L. Gao, D. Li, A. L. Pan, S. M. Li, *Sci Bull* **2024**, 69, 473.

[14]	K. C. Zhu, S. Pazos, F. Aguirre, Y. Q. Shen, Y. Yuan, W. W. Zheng, O. Alharbi, M. A. Villena, B. Fang, X. Y. Li, A. Milozzi, M. Farronato, M. Muñoz-Rojo, T. Wang, R. Li, H. Fariborzi, J. B. Roldan, G. Benstetter, X. X. Zhang, H. N. Alshareef, T. Grasser, H. Q. Wu, D. Ielmini, M. R. Lanza, *Nature* **2023**, 618, 57.

[15]	L. F. Sun, Z. R. Wang, J. Jiang, Y. Kim, B. Joo, S. Zheng, S. Lee, W. J. Yu, B. S. Kong, H. Yang, *Sci Adv* **2021**, 7.

[16]	D. Xiang, T. Liu, J. L. Xu, J. Y. Tan, Z. H. Hu, B. Lei, Y. Zheng, J. Wu, A. H. C. Neto, L. Liu, W. Chen, *Nat Commun* **2018**, 9.

[17]	J. Lee, S. Pak, Y. W. Lee, Y. Cho, J. Hong, P. Giraud, H. S. Shin, S. M. Morris, J. I. Sohn, S. Cha, J. M. Kim, *Nat Commun* **2017**, 8.

[18]	a) T. Ahmed, M. Tahir, M. X. Low, Y. Y. Ren, S. A. Tawfik, E. L. H. Mayes, S. Kuriakose, S. Nawaz, M. J. S. Spencer, H. Chen, M. Bhaskaran, S. Sriram, S. Walia, *Adv Mater* **2021**, 33; b) D. D. Kumbhar, Y. Je, S. G. Hong, D. H. Y. Lee, H. Kim, M. J. Kwon, S. Y. Cho, D. H. Lee, D. H. Lim, S. Kim, J. H. Park, *Adv Funct Mater* **2024**, 34; c) Y. K. Zheng, S. Ghosh, S. Das, *Adv Mater* **2024**, 36.

[19]	a) A. Dodda, D. Jayachandran, S. S. Radhakrishnan, A. Pannone, Y. K. Zhang, N. Trainor, J. M. Redwing, S. Das, *Acs Nano* **2022**, 16, 20010; b) T. R. Lin, L. C. Shih, P. J. Cheng, K. T. Chen, J. S. Chen, *Rsc Adv* **2020**, 10, 42682; c) M. Kumar, J. Lim, S. Kim, H. Seo, *Acs Nano* **2020**, 14, 14108; d) T. Ohno, T. Hasegawa, T. Tsuruoka, K. Terabe, J. K. Gimzewski, M. Aono, *Nat Mater* **2011**, 10, 591; e) M. Huang, W. Ali, L. L. Yang, J. H. Huang, C. D. Yao, Y. F. Xie, R. H. Sun, C. G. Zhu, Y. K. Tan, X. Liu, S. M. Li, Z. W. Li, A. L. Pan, *Adv Sci* **2023**, 10.

[20]	M. K. Rajbhar, D. Kumar, H. R. Li, D. D. Kumbhar, A. Singh, A. M. Syed, M. H. R. Ansari, S. Tytov, B. Alqahtani, H. Lee, N. El-Atab, *Mat Sci Eng R* **2026**, 167.

[21]	a) P. Giannozzi, S. Baroni, N. Bonini, M. Calandra, R. Car, C. Cavazzoni, D. Ceresoli, G. L. Chiarotti, M. Cococcioni, I. Dabo, *Journal of physics: Condensed matter* **2009**, 21, 395502; b) P. Giannozzi, O. Andreussi, T. Brumme, O. Bunau, M. B. Nardelli, M. Calandra, R. Car, C. Cavazzoni, D. Ceresoli, M. Cococcioni, *Journal of physics: Condensed matter* **2017**, 29, 465901.

[22]	Y. K. Zhang, W. T. Yang, *Phys Rev Lett* **1998**, 80, 890.

[23]	G. Orchard, A. Jayawant, G. K. Cohen, N. Thakor, *Front Neurosci-Switz* **2015**, 9.

[24]	W. Fang, Z. F. Yu, Y. Q. Chen, T. Masquelier, T. J. Huang, Y. H. Tian, *2021 Ieee/Cvf International Conference on Computer Vision (Iccv 2021)* **2021**, DOI: 10.1109/Iccv48922.2021.002662641.
